# The human intermediate prolactin receptor is a mammary proto-oncogene

**DOI:** 10.1038/s41523-021-00243-7

**Published:** 2021-03-26

**Authors:** Jacqueline M. Grible, Patricija Zot, Amy L. Olex, Shannon E. Hedrick, J. Chuck Harrell, Alicia E. Woock, Michael O. Idowu, Charles V. Clevenger

**Affiliations:** 1 Department of Pathology and Massey Cancer Center, Virginia Commonwealth University, Richmond, VA USA; 2 Department of Human and Molecular Genetics, Virginia Commonwealth University, Richmond, VA USA; 3 Wright Center for Clinical and Translational Research, Virginia Commonwealth University, Richmond, VA USA

**Keywords:** Tumour biomarkers, Oncogenes

## Abstract

The hormone prolactin (PRL) and its receptor (hPRLr) are significantly involved in breast cancer pathogenesis. The intermediate hPRLr (hPRLrI) is an alternatively-spliced isoform, capable of stimulating cellular viability and proliferation. An analogous truncated mouse PRLr (mPRLr) was recently found to be oncogenic when co-expressed with wild-type mPRLr. The goal of this study was to determine if a similar transforming event occurs with the hPRLr in human breast epithelial cells and to better understand the mechanism behind such transformation. hPRLrL+I co-expression in MCF10AT cells resulted in robust in vivo and in vitro transformation, while hPRLrI knock-down in MCF7 cells significantly decreased in vitro malignant potential. hPRLrL+I heterodimers displayed greater stability than hPRLrL homodimers, and while being capable of activating Jak2, Ras, and MAPK, they were unable to induce Stat5a tyrosine phosphorylation. Both immunohistochemical breast cancer tissue microarray data and RNA sequencing analyses using The Cancer Genome Atlas (TCGA) identified that higher hPRLrI expression associates with triple-negative breast cancer. These studies indicate the hPRLrI, when expressed alongside hPRLrL, participates in mammary transformation, and represents a novel oncogenic mechanism.

## Introduction

The polypeptide hormone prolactin (PRL) and its cognate receptor (hPRLr) are required for normal alveolo-/lacto-genesis^1^. hPRLr, a member of the type I cytokine receptor family, exists pre-dimerized at the cell surface where it is activated by ligand stimulation^2^. At the cellular level, PRL stimulates proliferation, migration, and viability, both in vitro and in vivo^1^. These physiologic effects are mediated by such signaling pathways as Janus kinase 2 (Jak2), signal transducer and activator of transcription 5a (Stat5a), phosphoinositide 3-kinase (PI3K), Src, mitogen-activated protein kinase (MAPK), and Ras, among many other signaling effectors^3,4^. While the actions of PRL in normal mammary gland development are well-known, its role in malignancy remains a point of debate. For example, while epidemiologic studies demonstrate a positive correlation between circulating PRL concentration and breast cancer risk, patients with hyperprolactinemia are overall not at an increased risk for breast cancer^5^. However, this discrepancy is likely a result of the marked decrease in estrogen production, which can be attributed to the hyperprolactinemic state^5^. Another root for this uncertainty is due to the close association of PRL with mammary differentiation. While the dichotomous actions of PRL in the normal mammary gland versus breast cancer (e.g., “milk versus malignancy”) are yet to be fully resolved, substantial evidence exists for PRL/hPRLr involvement in tumorigenesis. Aberrant PRL signaling has been shown to contribute significantly to breast cancer pathogenesis, and as stated prior, elevated serum PRL concentrations correlate positively with breast cancer risk^1^. Oncomine cancer microarray and immunohistochemical (IHC) analyses have demonstrated that hPRLr expression increases as a function of malignancy, and transgenic rodent studies show that PRL over-expression is sufficient for spontaneous tumorigenesis^6,7^. Furthermore, pan-hPRLr knock-down (KD) has been shown to be sufficient to inhibit cellular proliferation and colony-forming potential of breast cancer cells^7^.

While the full-length hPRLr (hPRLrL) form has traditionally been the focus of study, the hPRLr exists as a number of isoforms, each of which behave uniquely in terms of ligand binding, downstream signaling capabilities, and ultimate physiologic effect^8–11^. Protein and mRNA analyses have also demonstrated variable tissue expression of these isoforms, independent of one another, suggesting distinct biological roles^9,10^. Furthermore, hPRLr isoforms are capable of heterodimerizing, contributing to the heterogenous effect of PRL on its target tissue^2,12^.

Recently, truncated mouse PRLr (mPRLr) mutants were found to promote tumor growth in mouse embryonic fibroblasts (MEFs)^13^. Whole genome sequencing (WGS) of spontaneous estrogen receptor alpha positive (ERα + ) Stat1^−/−^ mammary tumors identified an mPRLr mutation hotspot (Supplementary Fig. 1a), leading to recurrent C-terminally truncated mPRLr mutants (mPRLrT; identified in 100% of sequenced primary tumors, and 0% of adjacent normal samples)^13^. The presence of truncating mPRLr mutations in 77.8% of ductal carcinoma in situ (DCIS) samples tested suggested mPRLrT involvement in tumor initiation^13^. All mPRLrT mutations were heterozygous at the allelic level, leading to the hypothesis that mPRLrT co-expression with wild-type long mPRLr (mPRLrL) was sufficient for tumorigenesis. Both in vitro and in vivo functional analyses confirmed that mPRLrL+T co-expression was sufficient for MEF transformation^13^. While compelling, these findings were not corroborated in a human mammary cell line, nor was a mechanism of transformation fully elucidated. Furthermore, analyses using both The Cancer Genome Atlas (TCGA) and Exome Aggregation Consortium (ExAC) confirmed that corresponding truncating mutations of hPRLr in breast cancer patients are rare (allele frequency <0.00001)^13^.

However, the mPRLrT shares significant homology with a hitherto under-studied, yet widely-expressed hPRLr isoform, the intermediate hPRLr (hPRLrI; Supplementary Fig. 1). hPRLrI, initially cloned from breast cancer patient samples and named for its resemblance to the intermediate rat PRLr, is generated by an out-of-frame splicing event^8,14^. This differential splicing event removes 79% of the hPRLr intracellular C-terminal domain, which contributes to ligand-stimulated signal transduction^8,10^. Some of the key residues lost as a result of the alternative splicing event include the phosphodegron serine 349 (S349), which is necessary for hPRLr protein degradation, as well as tyrosines 509 and 587 (Y509 and Y587, respectively), both of which have been implicated in PRL-driven canonical Stat5a activation^15–17^. The subdomain necessary for Jak2 interaction, namely the conserved Box 1 region, however, is retained^3^. Initial hPRLrI characterization demonstrated this isoform is capable of enhancing both cellular viability and proliferation, when expressed on its own in the murine myeloid cell line Ba/F3^10^. hPRLrI was also found to activate Jak2 signaling, but not that of Fyn, and transcript expression analyses confirmed hPRLrI tissue expression is unique from that of hPRLrL^10^. These preliminary data suggest a unique physiologic role for hPRLrI, independent of hPRLrL.

Considering the degree of sequence similarity between hPRLrI and mPRLrT, we hypothesized that hPRLrL+I co-overexpression in normal mammary epithelia would be sufficient for malignant transformation. This hypothesis parallels the actions of Her2 in breast cancer, wherein hPRLrI would require its full-length hPRLrL partner to achieve its full oncogenic potential^18^. Within this study, putative mechanisms of transformation were also examined, with a focus on differential heterodimeric versus homodimeric complex stability and signaling capabilities.

## Results

### hPRLrL + I co-expression in MCF10AT cells promotes malignant transformation, both in vivo and in vitro

**Fig. 1:**
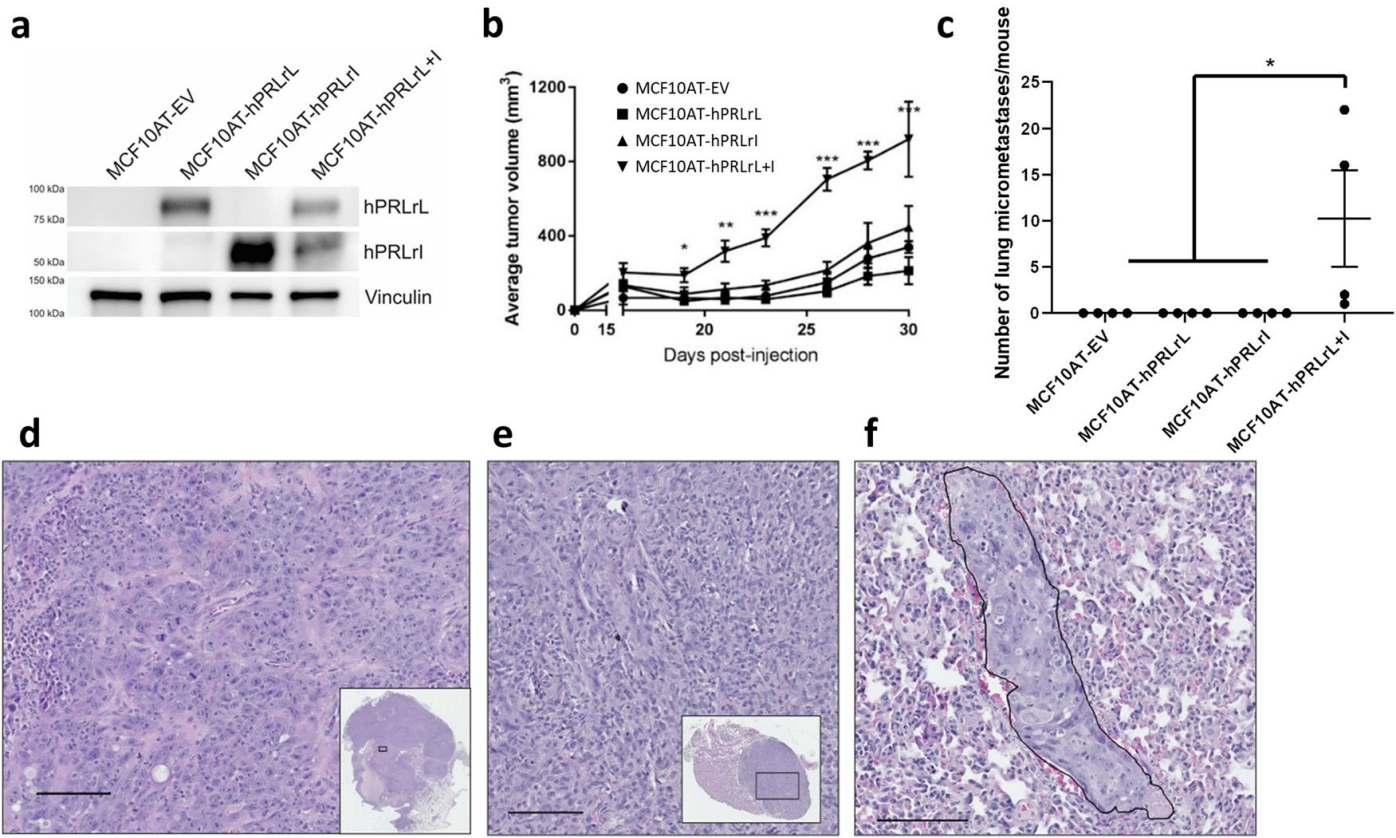
MCF10AT hPRLrL + I co-expression in vivo. **a** MCF10AT cells were stably transfected with either hPRLrL, hPRLrI, both isoforms together (at a 1:1 ratio), or empty vector (MCF10AT-hPRLrL, MCF10AT-hPRLrI, MCF10AT-hPRLrL+I, and MCF10AT-EV, respectively), and expression was confirmed by IB. **b** These cells were orthotopically xenografted into female NSG mice, and primary tumor growth was monitored longitudinally **c** Lungs were harvested and micrometastatic burden was counted manually. Representative images (scale bar, 100 µm) of H&E stained **d** primary tumor, **e** axillary lymph node and **f** lung indicate primary tumors to be high grade adenocarcinoma with nodal infiltration and successful distant metastatic dissemination (outlined; representative image shows metastatic growth long a blood vessel). *p < 0.05, **p < 0.01, ***p < 0.005. *n* = 4 mice/study arm. Data is shown as mean ± SEM.

To test the hypothesis that hPRLrL+I co-expression is sufficient for mammary transformation, MCF10AT cells were stably transfected with either isoform individually (MCF10AT-hPRLrL, MCF10AT-hPRLrI), both isoforms together (1:1; MCF10AT-hPRLrL+I), or empty vector (MCF10AT-EV). MCF10AT cells are a partially transformed human breast basal epithelial cell line expressing oncogenic HRAS (G12V), which when xenografted into immunocompromised mice have an invasive carcinoma rate of approximately 25%, and do not metastasize^19^. This cell line was chosen due to both its low level of endogenous hPRLrL and hPRLrI expression (Fig. 1a) as well as its low level of intrinsic tumorigenic potential. Following stable transduction, respective hPRLr isoform expression was confirmed by immunoblot (IB) (Fig. 1a). These MCF10AT transfectants were then orthotopically and bilaterally xenografted using female NSG mice. The mice harboring MCF10AT-hPRLrL+I transfectants developed primary tumors significantly more readily than empty vector, hPRLrL, or hPRLrI xenografts (Fig. 1b). Additionally, the primary tumors that formed from MCF10AT-hPRLrL+I xenografts grew more quickly than the other three study arms (Fig. 1b). Microscopic examination of harvested lungs revealed significant micrometastatic burden in 100% of the hPRLrL+I xenografts, which was not observed in the other three study groups (Fig. 1c, f). Histologic examination revealed MCF10AT-hPRLrL+I primary tumors were high grade adenocarcinoma (Fig. 1d) with demonstrable axillary lymph node infiltrate (Fig. 1e). Immunohistochemical (IHC) analyses confirmed these tumors were ER-/PR-/Her2- with high Ki67 status (Supplementary Fig. 2).

**Fig. 2:**
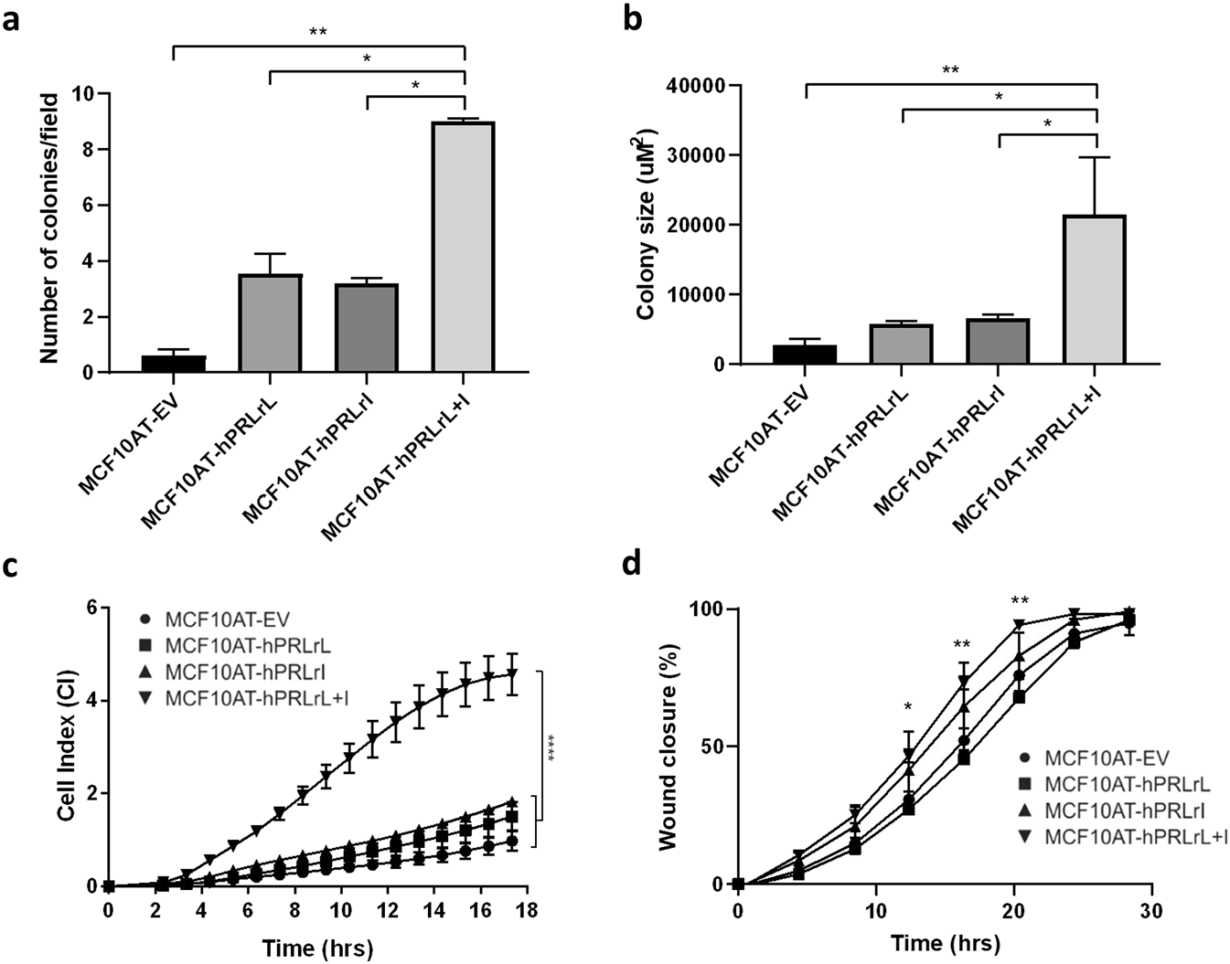
MCF10AT hPRLrL + I co-expression in vitro. MCF10AT over-expression transfectants were grown in soft agar and both **a** colony number and **b** colony size were quantified using CellProfiler. **c** Proliferation was measured using an xCELLigence apparatus, and **d** migration was assayed for via wound closure using an Incucyte® system. *p < 0.05, **p < 0.01, ****p < 0.001, *n* = 3. Data is shown as mean ± SEM.

To corroborate and assess the biological significance of hPRLrL+I co-expression in vitro, these MCF10AT transfectants were analyzed for the ability to grow in anchorage-independent conditions using soft agar. Cells co-expressing both hPRLr isoforms formed more colonies in soft agar than those expressing either hPRLrL, hPRLrI, or empty vector alone (Fig. 2a). Additionally, MCF10AT-hPRLrL+I colonies were overall significantly larger than those of the other three transfectants (Fig. 2b). Differential effect on both proliferation and migration were also examined, given the well-characterized mitogenic and pro-motility activities of PRL^1^. Analyses revealed that in vitro hPRLrL+I co-expression provides both a proliferative (Fig. 2c) and migratory (Fig. 2d) advantage, significantly above that provided by individual isoform expression. These data support the in vivo findings (Fig. 1b, c), indicating hPRLrL+I co-expression provides both proliferative and migratory advantages, both in vitro and in vivo.

These experiments were then repeated in the parental fully-nontransformed MCF10A cell line. While the in vitro results were similar to that observed with MCF10AT (Supplementary Fig. 3), MCF10A transfectants were unable to form tumors in NSG mice (data not shown). These data suggest a possible in vivo cooperative role for RAS in the observed tumorigenic potential. These results, taken as a whole, indicate hPRLrL+I co-expression promotes both in vivo tumor formation and metastatic spread as well as in vitro cellular transformation. Additionally, to determine the importance of the hPRLrL:hPRLrI ratio in in vitro proliferation, MCF10A cells were stably transfected to express varying levels of hPRLrL:hPRLrI protein (5:1, 2:1, 1:1, 1:2, and 1:5). It was observed that MCF10A cells expressing a 2:1 and 1:1 hPRLrL:hPRLrI ratio proliferated significantly faster than the other three conditions (data not shown).

### Knock-down of hPRLrI from MCF7 cells reduces colony forming potential and slows proliferation in vitro

**Fig. 3:**
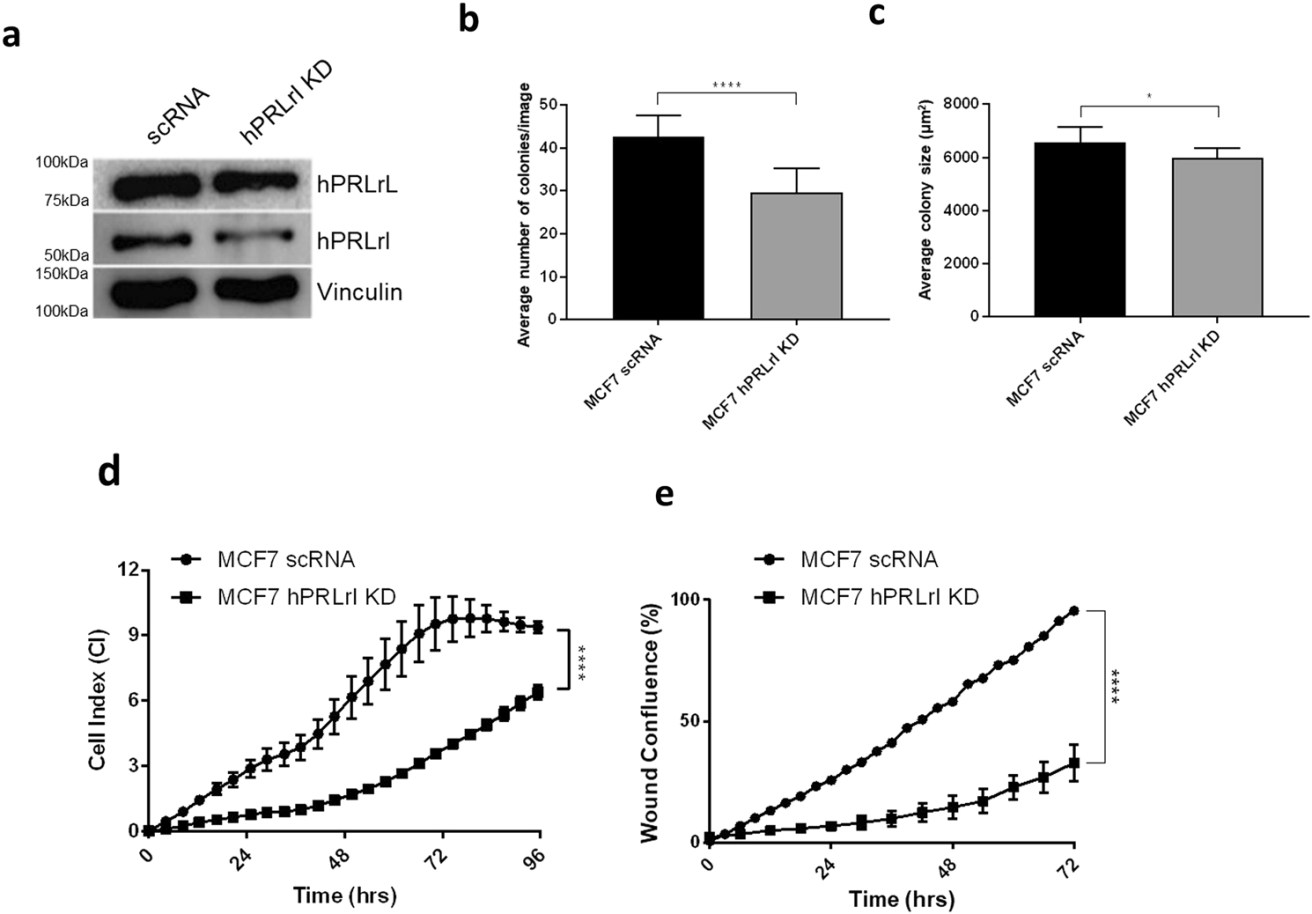
In vitro knock-down of hPRLrI in MCF7 cells. **a** MCF7 cells were stably transfected with either scrambled RNA (scRNA) negative control or shRNA targeting the hPRLrI splice site. These transfectants were analyzed for differential anchorage-independent growth via soft agar assay, quantifying both **b** colony number and **c** colony size, using CellProfiler. Transfectants were further assessed for **d** proliferative potential and **e** ability to migrate. *p < 0.05, ****p < 0.001, *n* = 3. Data is shown as mean ± SEM.

While hPRLr KD has been shown to significantly abrogate tumorigenic potential of breast cancer cell lines, studies to date have primarily focused on either pan-hPRLr or hPRLrL-specific KD^7,20^. Furthermore, given that hPRLrI is expressed in a wide array of breast cancer cell lines (Supplementary Fig. 4a), patient-derived xenografts (PDX; Supplementary Fig. 4b), and is not detectable at the protein level in either normal breast MCF10A cells or the partially-transformed derivative MCF10AT (Supplementary Fig. 4c, Fig. 1a), the effect of hPRLrI loss on breast cancer cells was examined in order to validate the oncogenic significance of this isoform in a more progressive stage of the disease^21^. To this end, isoform-specific KD was performed using MCF7 cells, which is an ER + luminal breast cancer cell line. This particular line was chosen given its moderate expression of both hPRLrI and hPRLrL (Supplementary Fig. 4A). Two shRNAs were designed to span the *hPRLrI* splice junction, which is unique to this isoform and would not co-hybridize to *hPRLrL* (see Materials and Methods). KD efficiency was confirmed by IB, and following stable transduction, a 43% reduction in hPRLrI protein expression was observed (Fig. 3a).

To determine the specific role of hPRLrI in mammary transformation, independent of that of hPRLrL, ability to grow in an anchorage-independent manner was examined. hPRLrI KD significantly decreased both the average number of colonies (Fig. 3b) as well as the size of colonies formed in soft agar (Fig. 3c), indicating hPRLrI is involved in the clonogenicity of breast cancer. Given than pan-hPRLr KD has been shown to hinder the proliferative potential of MCF7 cells, both differential proliferation and migration following hPRLrI KD were also assessed^7^. It was observed that MCF7-hPRLrI KD cells both proliferate slower (Fig. 3d) and migrate less readily (Fig. 3e) than MCF7-scRNA controls. Additionally, hPRLrI over-expression in the KD transfectants was sufficient to rescue the malignant phenotype (Supplementary Fig. 5). These experiments were repeated using an additional ER + luminal breast cancer cell line, T47D, and a similar phenotype was observed (Supplementary Fig. 6). Overall, these experiments indicate hPRLrI contributes to the malignant phenotype of both anchorage-independent and anchorage-dependent growth of breast cancer cells.

### hPRLrL + I heterodimers are more stable than hPRLrL homodimers

**Fig. 4:**
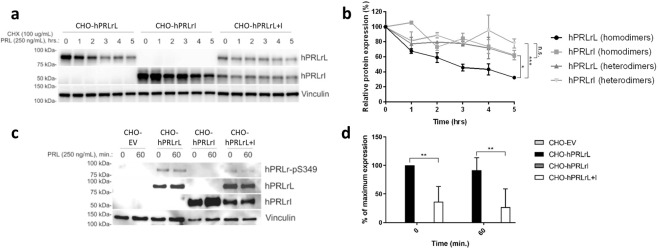
hPRLr heterodimer versus homodimer differential stability. Chinese hamster ovary (CHO) cells (PRLr-null) were transiently-transfected with either hPRLrL, hPRLrI, or both isoforms concurrently (at a 1:1 ratio). **a** Transfectants were simultaneously stimulated with cycloheximide (CHX; 100 µg/mL) and prolactin (PRL; 250 ng/mL), and lysates were harvested once per hour for 5 consecutive hours. Protein degradation was quantified, via densitometry of band intensity, in **b** and t_1/2_ was extrapolated from each respective slope. **c** Phosphorylation status of the hPRLr phosphodegron was assessed using a pS349-specific antibody (gift of Dr. Serge Fuchs), and **d** band intensity was quantified via densitometry. *p < 0.05, **p < 0.01, ***p < 0.005, *n* = 3. Data is shown as mean ± SEM.

Given these data supporting the hypothesis that hPRLrI is a proto-oncogene, assessment of potential transforming mechanisms was carried out. In previous studies, expression of a degradation-resistant hPRLr was able to accelerate both proliferation, invasion, and tumorigenic potential^15,22,23^. Furthermore, the cryptic splicing event which generates hPRLrI removes the phosphodegron S349 from the receptor (Supplementary Fig. 1b). Therefore, to determine if the loss of S349 from hPRLrI effects hPRLr homodimer and/or heterodimer stability, hPRLr complex half-life (t_1/2_) was directly assayed using a cycloheximide (CHX) approach. Chinese hamster ovary (CHO) cells (PRLr-null) were transiently transfected with either empty vector (CHO-EV), each isoform individually (CHO-hPRLrL and CHO-hPRLrI, respectively), or both concurrently (CHO-hPRLrL+I). Following dual PRL/CHX treatment, hPRLrL homodimers were found to have a t_1/2_ of approximately 3 h, as is consistent with literature reports (Fig. 4a, b)^23^. Conversely, both hPRLrL+I heterodimers and hPRLrI homodimers were discovered to have a t_1/2_ of 7.5 h, indicating these complexes containing the intermediate isoform are significantly more stable than their full-length counterpart (Fig. 4a, b). These data indicate that hPRLrL degradation is significantly impaired following heterodimerization with hPRLrI, which may be the result of hPRLr-S349 phosphodeficiency. Therefore, as a corollary to these results, the phosphorylation status of S349 in these transfectants was assessed. Robust pS349-hPRLr expression was observed on hPRLrL homodimers, but this effect was found to be significantly mitigated on hPRLrL+I heterodimers (Fig. 4c, d). These results indicate that hPRLrL+I heterodimers are significantly more stable than that of hPRLrL homodimers, and that this increase in complex stability may be a contributing mechanism in the observed hPRLrL+I-driven mammary transformation.

### hPRLrL + I heterodimers are capable of stimulating Jak2, Ras, and MAPK activation, but do not stimulate Stat5A tyrosine phosphorylation

**Fig. 5:**
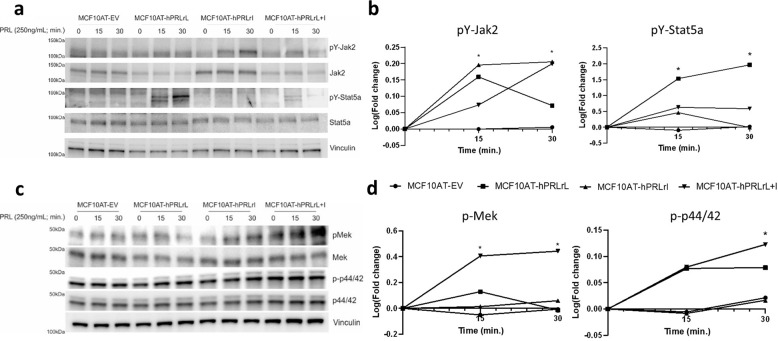
hPRLrL + I heterodimer signal transduction. MCF10AT transfectants were PRL (250 ng/mL) stimulated for 0, 15, and 30 min, and phospho-IB was utilized to assess the activation status of **a**, **b** Jak2/Stat5a and **c**, **d** Mek/Erk. Band intensities were quantified using densitometry. Significance was determined by comparing each condition to the MCF10AT-EV negative control for each respective time point. *p < 0.05, *n* = 3.

hPRLr stability has been shown to have a significant impact on the downstream signaling kinetics of the receptor, suggesting the observed hPRLrL+I increased t_1/2_ may augment hPRLr signal transduction^15^. PRL stimulation of hPRLr activates an intricate signaling network, involving such effectors as Jak2/Stat5a, MAPK, PI3K, Ras, and others^3,4^. Furthermore, previous reports indicate rat PRLr (rPRLr) heterodimers signal uniquely from homodimers, resulting in differential physiologic effect^12,24^. To assess the differential signaling properties of hPRLrL+I heterodimers, MCF10AT transfectants were stimulated with PRL, and the activation status of Jak2/Stat5a, Mek1/2, and Erk1/2 pathways were examined by phospho-IB. Following PRL stimulation, hPRLrL and hPRLrI homodimers, as well as hPRLrL+I heterodimers, were found to induce Jak2 tyrosine phosphorylation (pY-Jak2) (Fig. 5a, b). hPRLrL+I heterodimers and hPRLrL homodimers stimulated both Erk1/2 threonine/tyrosine phosphorylation (p-p44/42) and Mek1/2 serine phosphorylation (Fig. 5c, d), while hPRLrI alone failed to activate either protein (Fig. 5c, d). hPRLrI homodimers, which entirely lack the Stat5a putative docking residue(s), were notably incapable of tyrosine-phosphorylating Stat5a (pY-Stat5a) (Fig. 5a, b). Strikingly, however, hPRLrL+I heterodimers were unable to induce pY-Stat5a expression, as compared to that induced by hPRLrL alone (Fig. 5a, b). These results indicate that Stat5a docking onto hPRLr requires the dimeric presence of its binding residue, which is consistent with precedent data obtained using rPRLr heterodimeric chimeras^12^. Taken as a whole, these data confirm that hPRLrL+I heterodimers exhibit signal transduction which is unique from either hPRLrL or hPRLrI homodimers. Furthermore, considering the role of pY-Stat5a in attenuating the kinase activity of Jak2, these data suggest that the unchecked actions of Jak2 may also be a significant contributory mechanism for hPRLrL+I-driven mammary transformation, in addition to the observed elevation in complex stability^25^.

### Oncogenic KRAS overexpression contributes to the in vitro transforming potential of hPRLrL + I co-expression

**Fig. 6:**
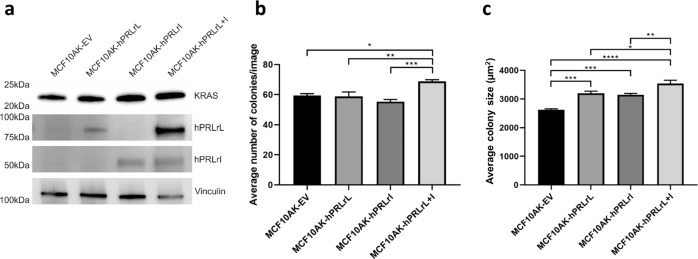
KRAS-G12V over-expression alongside hPRLrL + I co-expression in vitro. **a** MCF10A hPRLr over-expression transfectants received an additional stable transfection of KRAS-G12V, and expression was confirmed via IB. Cells were then grown in soft agar, and both **b** colony number and **c** colony size were determined. *p < 0.05, **p < 0.01, ***p < 0.005, ****p < 0.001. *n* = 3. Data is shown as mean ± SEM.

The differential in vivo results of hPRLr isoform overexpression in MCF10A versus MCF10AT cells (which harbor an oncogenic form of Ras; Fig. 1), suggest Ras may cooperate with hPRLrL+I heterodimers during transformation. Furthermore, recent studies with a transgenic PRL over-expression mouse model have implicated KRAS signaling involvement in PRL-dependent mammary neoplasias^6,26^. KRAS promotes metastatic dissemination, and therefore, is strongly correlated with worse overall patient outcome^27^. hPRLrL+I-driven activation of Erk1/2 and Mek1/2 (Fig. 5c, d), which are downstream signaling effectors of KRAS, suggests a pivotal role for this potent oncogene in the observed hPRLrL+I-driven cellular transformation. To test this hypothesis, a constitutively active KRAS mutant (G12V) was introduced into the above described MCF10A transfectants (hereafter referred to as MCF10AK) (Fig. 6a). These cells were then grown in soft agar to assess transforming potential in vitro. While oncogenic KRAS over-expression increased MCF10A basal colony forming potential (Supplementary Fig. 3d), expression of either hPRLr isoform alone did not increase clonogenicity above the empty vector negative control (Fig. 6b). However, a significant increase in colony forming potential was observed following hPRLrL+I co-expression, and these colonies were on average larger than those formed by MCF10AK-hPRLrL or MCF10AK-hPRLrI (Fig. 6c). These data suggest that KRAS works cooperatively with hPRLrL+I, to promote cellular transformation.

### hPRLrI expression increases with tumor grade, and is associated with high proliferative index and TNBC

The data presented above outline a novel role for hPRLrI as a breast cancer proto-oncogene. While multiple studies have highlighted the clinical relevance of hPRLr, no studies to date have examined the clinical significance of specifically hPRLrI in breast cancer. Isoform-specific protein studies of hPRLr remain a challenge owing in part to limited options in reagents. In order to address this issue and directly assay the clinical significance of hPRLrI, a novel polyclonal antibody (pAb) was generated against a portion of the I-Tail (Supplementary Fig. 1b), which is entirely unique to this isoform (Supplementary Fig. 7) (See Materials and Methods). This antibody displayed a high level of both isoform-specificity, for both IHC and IB purposes (Supplementary Fig. 7), and was subsequently used to assess the clinical relevance of hPRLrI via tissue microarray (TMA).

**Fig. 7:**
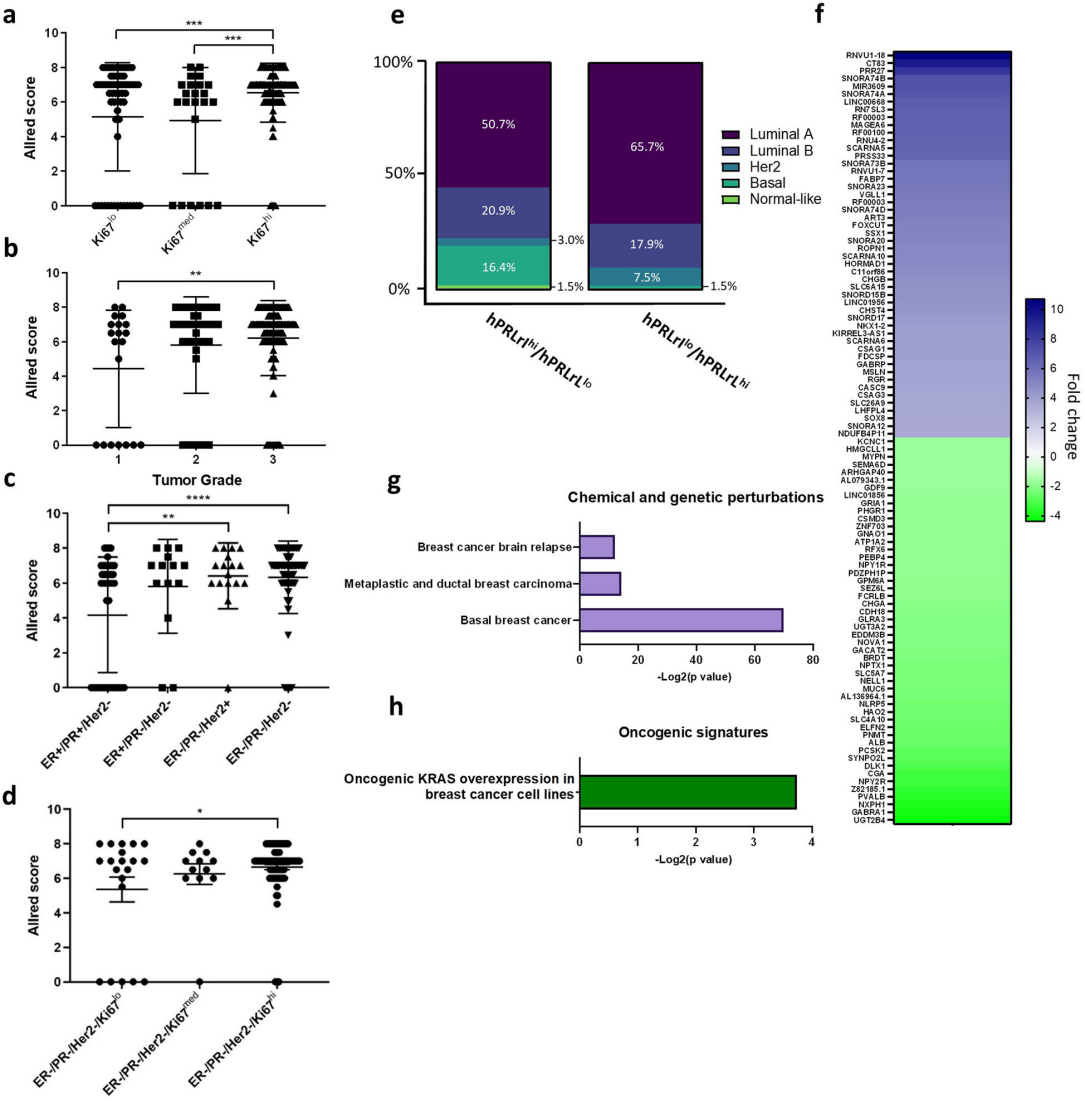
Protein and transcript expression of hPRLrI in breast cancer clinical samples. hPRLrI protein in breast cancer clinical samples (*n* = 250) was analyzed by tissue microarray (TMA) analysis. Allred score is reflective of both hPRLrI staining intensity and percent positivity. **a** Proliferative index, reported via Ki67 status, was assessed, as was **b** tumor grade and **c** hormone receptor status. **d** Proliferative index within the ER-/PR-/Her2- cohort was also analyzed. The hPRLrI:hPRLrL transcript ratio was also examined, using TCGA-reposited RNAseq data. **e** Breast cancer intrinsic subtype frequency was assessed for the hPRLrI^hi^/hPRLrL^lo^ versus hPRLrI^lo^/hPRLrL^hi^ ratio tertiles (*n* = 67/tertile). **f** Differential gene expression patterns were characterized, with the heatmap displaying the top/bottom 50 differentially-expressed genes (DEGs), comparing the top/bottom hPRLr isoform ratio tertile. These DEGs were then analyzed via GSEA, looking within the **g** chemical and genetic perturbation and **h** oncogenic signatures gene sets. *p < 0.05, **p < 0.01, ***p < 0.005, ****p < 0.001. Data is shown as mean ± SEM.

In order to characterize associations between hPRLrI protein expression and clinical characteristics, a total of 250 clinically-staged breast cancer patient samples were obtained, and hPRLrI protein expression was assayed via IHC. Following hPRLrI scoring using the Allred method (modified here to reflect hPRLrI cytoplasmic reactivity), it was discovered that hPRLrI expression increased with both proliferative index (Fig. 7a) as well as tumor grade (Fig. 7b)^28^. Furthermore, hPRLrI expression was found to be highest in those patients with TNBC (Fig. 7c), and that within the TNBC patient subset, hPRLrI expression increased with Ki67 status (Fig. 7d). While past studies have highlighted a significant role for hPRLr in ER + /PR + disease, the actions of this receptor and its cognate hormone in TNBC remain enigmatic and controversial^29,30^. These data highlight a heretofore novel role for hPRLr, specifically this splice variant, in TNBC.

### High hPRLrI:hPRLrL ratio is associated with aggressive, basal-like breast cancers, with significant enrichment in KRAS signaling

Given the evidence that greater hPRLrI protein expression associates with more aggressive tumors, and in consideration of the in vivo and in vitro results that indicate hPRLrI requires concurrent hPRLrL expression in order to meet its full malignant potential, in silico analyses of the cooperative oncogenic actions of hPRLrI with hPRLrL were assessed. To this end, RNA sequencing (RNAseq) data reposited within The Cancer Genome Atlas (TCGA) breast cancer (BRCA) cohort was mined for both *hPRLrI* and *hPRLrL* transcript expression. Considering that the observed cellular transformation was seen only when both isoforms were co-expressed, assessment of the hPRLrI:hPRLrL ratio was carried out using these transcript data. Consequently, an increase in the ratio of hPRLrI:hPRLrL was hypothesized to correlate with worse overall patient prognosis.

Considering the association observed between hPRLrI and TNBC in the TMA analysis (Fig. 7c), the ratio of *hPRLrI* to *hPRLrL* transcript expression was used to parse TCGA samples by breast cancer intrinsic molecular subtype^31^. Inasmuch, it was observed that those primary tumor samples with a high hPRLrI:hPRLrL ratio were more likely to be classified as basal-like breast cancer (Fig. 7e; Supplementary Table) when compared to samples with a low hPRLrI:hPRLrL ratio. These data were corroborated at the in vitro level, with basal-like model cell line MDA-MB-436, as well as the claudin-low cell lines SUM1315 and MDA-MB-231, possessing a high hPRLrI:hPRLrL ratio (Supplementary Fig. 4A). Furthermore, a higher hPRLrI:hPRLrL ratio was found to be associated with primary tumors that were overall larger in size and were more likely to be higher grade (Supplementary Table). These data corroborated the TMA findings, in which higher hPRLrI relative expression associated with more aggressive breast malignancies.

To further evaluate the actions of the hPRLrL+I complex, differentially-expressed gene (DEG) analyses were performed using these TCGA data, to compare those differences observed between the high versus low hPRLrI:hPRLrL ratio cohorts. A total of 345 genes were significantly enriched in the hPRLrI^hi^/hPRLrL^lo^ cohort, and 81 genes were concurrently under-represented (Fig. 7f). Gene-set enrichment analysis (GSEA) uncovered a significant association within the hPRLrI^hi^/hPRLrL^lo^ cohort with basal-like breast cancer, metastasis, and oncogenic KRAS actions (Fig. 7g, h). Additionally, within the enrichment dataset, a number of potent oncogenes were noted: namely, ROS1 and BCL11A, both of which have recently been implicated in TNBC^32,33^. In contrast, tumors with an hPRLrI^lo^/hPRLrL^hi^ ratio were enriched in genes associated with luminal breast cancer (e.g., CGA, PVALB, NOVA1; Fig. 7f), and were more likely to be small in size and early stage (Supplementary Table). While outcomes and racial diversity data in this cohort are currently limited, the significant association of a high hPRLrI:hPRLrL ratio with metastatic, basal-like breast cancer have implications for future disparities research. Taken together, these data corroborate both the TMA clinicopathologic data as well as the in vitro cooperative actions of KRAS in hPRLrL+I co-expression.

## Discussion

Although a role for PRL/hPRLr in breast cancer has been well-documented, efforts to tease out the functional and clinical significance of PRL in malignancy have largely been displaced by its role in normal physiology. One possible explanation for this dichotomy of “milk versus malignancy” may lie in the unique functions of the individual hPRLr isoforms, whose respective biological roles in breast cancer remain poorly understood. In this study, hPRLrI is identified as a novel breast cancer proto-oncogene that reaches its full oncogenic potential when expressed in concert with hPRLrL. As presented here, hPRLrL+I heterodimers: (1) significantly contribute to distant metastasis formation, (2) exhibit greater complex stability than their full-length counterpart, (3) display unique signal transduction, and (4) cooperate with KRAS signal transduction. These data suggest that hPRLrI oncogenicity acts in a manner similar to that of Her2: meaning, the full-length receptor partner (e.g., hPRLrL compared to Her1/3/4 for Her2) is necessary to achieve hPRLrI’s full oncogenic potential^18^. Similarly, the homologous intermediate form in rats (rPRLrI) has also been implicated in tumorigenesis, having been initially studied as a potential oncogene in the rat lymphoma cell line Nb2^14^.

As shown here, hPRLrL+I co-expression resulted in not only rapid primary tumor growth, but also significant lung micrometastatic burden, when acting in concert with oncogenic Ras. This observation was corroborated in analyzing TCGA breast cancer cohort DEG data, where a high hPRLrI:hPRLrL ratio associated with a greater likelihood of breast cancer brain relapse, distant metastasis, and KRAS activity. As data regarding survival outcomes and metastatic burden within both the TCGA-BRCA cohort and the breast cancer TMA used are limited, future studies will be necessary to fully parse the unique role of hPRLrI in breast cancer metastasis, racial disparities, and survival outcomes. Additionally, hPRLr was recently implicated in being a clinical marker for metastatic risk, however literature examining hPRLr isoform-specific actions is still lacking^34^.

Protein degradation assays revealed hPRLrL+I heterodimers are significantly more stable than hPRLrL homodimers. These results are of particular relevance given the well-documented oncogenic effect of increased hPRLr stability in breast cancer^15,22,23,35^. Elevated hPRLr stability results in augmented signal transduction, leading to greater cellular proliferation, anchorage-independent growth, and tumorigenicity^15^. Phospho-IB also confirmed that this increase in complex t_1/2_ correlated with a decrease in hPRLr-S349 phosphorylation status, suggesting that monomeric hPRLr-S349 expression is insufficient for PRL-induced phosphorylation at S349. This phosphodeficiency would thereby prevent recruitment of the ubiquitin-ligase machinery to the receptor and subsequent protein turnover^15^.

hPRLr signal transduction is directly influenced by receptor stability. hPRLrL+I heterodimers are incapable of stimulating Stat5a tyrosine phosphorylation, which is required in the normal mammary gland for development and milk production. To this end, unphosphorylated Stat5a (upY-Stat5a) has been dogmatically thought of as inactive^36,37^. However, emerging data with both upY-Stat3 and upY-Stat5a have indicated that upY-Stats are indeed active and function independently from their phosphorylated counterpart^38,39^. Notably, overexpression of phosphodeficient Stat5a-Y694F in HeLa cells was recently shown to promote and stabilize heterochromatin formation, which was sufficient for global transcriptomic downregulation^38^. Many of the genes epigenetically silenced by upY-Stat5a carry tumor suppressive functions (e.g., TIMP4, SH3GL2), suggesting upY-Stat5a itself may be involved in hPRLrL+I-driven mammary transformation^38^. However, the role of Stat5a in breast cancer remains unsettled: elevated pY694-Stat5a in clinical samples associates with more differentiated tumors and better prognosis, yet Stat5a knock-out mice display delayed tumor onset^40,41^. One possible explanation for this discrepancy involves the actions of Stat5a in regulating Jak2 activity. pY694-Stat5a facilitates attenuation of Jak2 kinase activity, indicating the apparent Stat5a dichotomy may be the result of unchecked Jak2 actions^25^. These results imply that a paucity of pY694-Stat5a activity, in conjunction with other active hPRLr signaling effectors (e.g., MAPK and Ras) may be contributory mechanistic factors in hPRLrL+I-driven transformation.

KRAS holds a prominent cooperative role with hPRLr in breast cancer pathogenesis. For example, in characterizing a PRL-driven mammary cancer mouse line (NRL-PRL), genomic analyses uncovered consistent oncogenic KRAS somatic mutations/amplifications in all primary tumors, which was not found in pre-neoplastic glands^26^. These findings coincided with recent discoveries of significant KRAS involvement in breast cancer metastatic dissemination, mirroring the in vivo results presented herein^27^. One possible mode of interplay between these two pathways may lie in the role of KRAS as a negative regulator of glycogen synthase kinase-3β (GSK3β), which itself is a negative regulator of hPRLr stability^42^. In this manner, KRAS activity indirectly stabilizes hPRLr, uncovering a possible mechanism for the observed cooperation between these two pathways in in vitro transformation.

While clinical analyses have successfully confirmed the relevance of hPRLr in breast cancer, limited options in reagents have hampered researchers in parsing out the specific effects of individual hPRLr isoforms^7^. Accordingly, the approaches utilized in this study were able to successfully focus on those clinical features that associated with both 1) hPRLrI expression, and 2) the hPRLrI:hPRLrL ratio. While a handful of recent studies have suggested a role for this receptor in TNBC, PRL/hPRLr have traditionally been associated with luminal breast cancers, and this association is observed with only hPRLrL^6,26,29^. Interestingly, the clinical data presented here highlight a novel association between the hPRLrI isoform and aggressive TNBC/basal-like breast cancers, corroborating both the in vivo and in vitro studies presented herein.

The association of hPRLr/PRL and breast cancer has garnered clinical interest, and a variety of pan-hPRLr antibodies have undergone phase I clinical trials^43^. These antibodies, all of which targeted the hPRLr-ECD which is fully conserved among the majority of annotated hPRLr isoforms (i.e. all but the ΔS1 isoform), were found to be safe yet clinically ineffective^9,43^. These agents lacked hPRLr isoform-specificity and, in consideration of the data presented here, would argue that future approaches in drug design may need to target hPRLrI specifically, which could ultimately serve as a unique breast cancer therodiagnostic.

## Methods

### Cell culture

MCF10A/MCF10AT cells were maintained in DMEM/F12 HEPES (11330032, ThermoFisher Scientific), supplemented with following additives at the given final concentration from the indicated sources: horse serum (5%, S1215OH, Atlanta Biologicals), hydrocortisone (0.5 mg/mL, H0888, Sigma-Aldrich), cholera toxin (100 ng/mL, C8052, Sigma-Aldrich), insulin (10 µg/mL, I0516, Sigma-Aldrich), penicillin/streptomycin (1%, 15140–122, ThermoFisher Scientific), and EGF (20 ng/mL, PHG0311L, ThermoFisher Scientific). MCF7/T47D/293 T cells were maintained in DMEM (21041025, ThermoFisher Scientific), supplemented fetal bovine serum (FBS; 10%, MT35011CV, Corning) and penicillin/streptomycin (1%, 15140–122, ThermoFisher Scientific). CHO cells were grown in Ham’s F-12K (Kaighn’s) Medium (21127022, ThermoFisher Scientific), with FBS (10%, MT35011CV, Corning) and penicillin/streptomycin (1%, 15140–122, ThermoFisher Scientific). For all serum-starvation studies, DMEM/F-12, no phenol red (21041025, ThermoFisher Scientific) was supplemented with bovine serum albumin (0.1%, 10735078001, Sigma-Aldrich). All cell lines were purchased through ATCC.

### Stable cell line generation

hPRLrL and hPRLrI, respectively, were cloned into the retroviral pBabe-GFP (10668, Addgene) backbone and used to make viral particles as previously described^7^. Briefly, 293 T cells were grown to 80% confluency in a 6-well dish and transfected (Lipofectamine^TM^; L3000015, ThermoFisher Scientific) with: 12ug retroviral construct, 8 μg pMLV-gag-pol, and 4ug pVSVG. Cells were incubated in OptiMEM (31985070, ThermoFisher Scientific)/transfection reagent overnight at 37 °C. At 24 h, the OptiMEM was replaced with fresh growth media, and cells were incubated at 32 °C for 16–20 h. The supernatant containing virus was harvested and immediately applied directly onto cells being transfected. Cells were “spin-fected” with the viral supernatant for 2 h at 500 *g*, 32 °C. Successfully infected cells were then selected either by fluorescence-activated cell sorting (FACS) or puromycin resistance (0.5 µg/mL) as appropriate. For oncogenic KRAS over-expression studies, KRAS G12V in pBabe-puro was generously gifted by Dr. Azeddine Atfi (Virginia Commonwealth University, VCU).

### Cloning

hPRLrL and hPRLrI, respectively, were cloned into the mammalian expression vector pTracer EF V5-HisA (V88720, Addgene) using the following procedure: total RNA was harvested from T47D cells using TRIzol^TM^ Reagent (15596026, ThermoFisher Scientific) per the manufacturer’s instructions. cDNA conversion was carried out using the iScript^TM^ cDNA synthesis kit (1708891, Bio-Rad) per the manufacturer’s protocol. hPRLrL and hPRLrI, respectively, were amplified using the following primers: HPRLR-Kpn1 (5′-GGACGGTACCCACCATGAAGG-3′), HPRLRL-Xho1 (5′-GCGCTCGAGGTGAAAGGAGTGTGTAAA-3′), and HPRLRI-Xho1 (5′-GCGCTCGAGGGAGTCCCGGGCTTC-3′), which correspond to the shared hPRLr ORF start position, hPRLrL stop position, and hPRLrI stop position, respectively. The PCR cycling was as follows: 98 °C for 30 s, 98 °C for 10 s, 64 °C for 30 s, 72 °C for 30 s/kb, these former three steps were repeated for 35 total cycles, followed by 2 min at 72 °C and a 4 °C hold. Amplicons were run on a 1% agarose gel, and band extraction, isolation, and purification was performed using the E.Z.N.A. Gel Extraction Kit (101318–970, Omega Bio-tek). Purified amplicons were sequenced by Eurofins (Luxembourg). Both insert and vector were double-digested with Kpn1 and Xho1, and the vector was treated with recombinant shrimp alkaline phosphatase (rSAP; M0371S, NEB) followed by heat inactivation. Insert and vector were ligated using T4 DNA ligase (M0202S, NEB), and transformed into One Shot® TOP10 Chemically Competent cells (C404003, ThermoFisher Scientific). Construct DNA was harvested using Qiagen’s MaxiPrep kit (12362).

### Transient transfection

All transient transfections of CHO cells utilized Lipofectamine 3000 Transfection Reagent (ThermoFisher Scientific), per the manufacturer’s instructions. For hPRLrL+I isoform co-expression, cells were simultaneously co-transfected at a 1:1 hPRLr isoform ratio.

### Mice

All studies described were approved by the VCU Institutional Animal Care and Use Committee (IACUC). Female 5–7 week old NSG mice were obtained from the VCU Cancer Mouse Model Core or Dr. J. Chuck Harrell (VCU). Mice were subject to bilateral intraductal injection of the 4^th^ set of mammary glands with MCF10AT hPRLr isoform over-expression transfectants (2.0×10^6^ cells/gland). Once tumors were palpable (approximately 10 mm^3^), tumors were calipered thrice weekly until total tumor burden was ≤2 cm^3^. At endpoint, mice were euthanized by CO_2_ asphyxiation followed by cervical dislocation. Following euthanasia, all primary tumors were harvested: one full tumor per mouse was subject to formalin fixation and paraffin embedding (FFPE), while the other was bifurcated with one half being snap frozen in liquid nitrogen and the other stored in RNA*later*^TM^ (AM7020, ThermoFisher Scientific) for future studies. Additionally, all lungs and axillary lymph nodes were harvested, subject to FFPE, and examined for micrometastases.

### Immunohistochemistry

Tissue fixation, dehydration, paraffin embedding, and hematoxylin and eosin (H&E) staining were performed as previously described^44^. Briefly, harvested tissues were fixed in 10% formalin, dehydrated, paraffin embedded. Five-micron sections were stained with the custom αhPRLrI primary antibody (New England Peptide) at a 1:12,500 titer, following an additional 10% formalin fixation blocking step prior to antigen retrieval. After washing, slides were incubated with SignalStain Boost IHC Detection Reagent (8114 S, Cell Signaling) for 1 h, followed by incubation with SignalStain DAB Substrate (8059 S, Cell Signaling) for 1 min. Slides were counterstained with hematoxylin (Gill No. 3; GHS332, Sigma), dehydrated, and coverslipped using Permount mounting medium (50–277–98, Electron Microscopy Sciences). hPRLrI staining was expressed as an Allred score, namely the sum of the proportion of positive cells (0–5 scale) and mean intensity (0–3 scale)^44^.

### Immunoblot

For all IB applications, cells were grown to approximately 80% confluency, washed with ice-cold 1x PBS, and harvested in RIPA (1% NP40, 1% sodium deoxycholate, 0.1% SDS, 0.15 M NaCl, 0.01 M sodium phosphate pH 7.2, 2 mM EDTA, 50 mM sodium fluoride) supplemented with 5% BME and 1x Laemmli Sample Buffer (161–0747, Bio Rad). For all PRL stimulation experimental lysates, cells were serum-starved for 16–20 h and stimulated at 250 ng/mL PRL (a physiologic level during pregnancy). All blots were derived from the same experiment and processed in tandem. Antibodies used for these studies were obtained from the following sources at the indicated titer: hPRLr ECD (35–9200, Invitrogen, 1:1000), pY-Stat5a (9359S, Cell Signaling, 1:1000), Stat5a (sc-1081, Santa Cruz Biotechnology, 1:1000), pY-Jak2 (3776S, Cell Signaling, 1:500), Jak2 (3230S, Cell Signaling, 1:500), p-p44/42 (9101S, Cell Signaling, 1:1000), p44/42 (9102S, Cell Signaling, 1:1000), pS-Mek (9121S, Cell Signaling, 1:1000), Mek (9122S, Cell Signaling, 1:1000), KRAS (14412S, Cell Signaling, 1:1000), hPRLrI (New England Peptide, 1:5000), pS349-hPRLr (Serge Y. Fuchs, M.D., Ph.D., University of Pennsylvania, 1:100), Vinculin (MCA465GA, Bio-Rad, 1:1000). Densitometry was performed using ImageQuant TL (GE) general image analysis software.

### Soft agar colony formation

Soft agar assays were performed as described previously^7^. Briefly, a 1 mL base layer of 0.6% noble agar/growth media was set in a 6-well plate. A 500uL overlay of 0.3% noble agar/growth media containing cells in a single-cell suspension (MCF10A/MCF10AT, 2.0×10^5^; MCF7/T47D, 1.0 × 10^5^) was seeded. Wells were overlaid with 1 mL growth media, which was replaced thrice weekly. Cells were cultured for 2 weeks, and colonies were stained using Thiazolyl blue tetrazolium bromide (MTT; AC15899-0010, ThermoFisher Scientific). 5–10 images per well at 4x magnification were obtained and quantified using CellProfiler (www.cellprofiler.org).

### Proliferation and migration

Proliferation was assessed using an xCELLigence apparatus (ACEA Biosciences Inc.). Cell Index (CI) provides a read-out of electrical impedance, which is applied in determining adherent cell density, allowing for real-time quantification of cellular proliferation. For MCF10A/MCF10AT experiments, 12,500 cells/well were seeded; MCF7, 5000 cells/well; T47D, 10,000 cells/well. Migratory potential was assayed using an Incucyte® (Sartorius) live-cell apparatus. For MCF10A/MCF10AT experiments, 40,000 cells/well were seeded; MCF7/T47D, 20,000 cells/well. Both proliferation and migration were assayed in complete growth media.

### hPRLrI isoform-specific knock-down

A pair of shRNA constructs were designed to target the hPRLrI splice junction, beginning at bp 1009 (RefSeq NM_001204315): 5′-CACCCAAGTCAAGAGAGAGAA-3′ and 5′-CCAAGTCAAGAGAGAGAACAG-3′. These oligomers were cloned into the psi-U6 vector (RSH050227-CU6, GeneCopoeia) and used to generate viral particles, as described above. These constructs were spinfected (see above) into MCF7 and T47D cells, and cells were sorted for high GFP expression. Cells were subsequently maintained in puromycin (0.5 μg/mL), and isoform-specific KD was confirmed via IB.

### hPRLrI polyclonal antibody generation

An hPRLrI-specific polyclonal antibody was generated by and purchased from New England Peptide (Gardner, MA). New Zealand White SPF rabbits were thrice immunized with a peptide corresponding to residues 331–346, located on the C-terminus of hPRLrI (Ac-C^331^KEHPSQEREQRQAQEA^346^-amide). Affinity purification was performed to yield the purified hPRLrI antibody (αhPRLrI). In order to confirm the specificity of the αhPRLrI pAb binding affinity, blocking peptide competition assays were performed for both IB and IHC approaches. Briefly, blocking peptide, corresponding to the antigen sequence used for inoculation in generating the antibody, was incubated alongside the primary antibody at a 50x excess to primary antibody concentration. This was followed by standard IB/IHC protocol (see above), followed by respective quantification of change in band/staining intensity (ImageQuant TL, GE).

### TCGA RNAseq analysis for hPRLr isoform-specific expression

RNAseq BAM files were downloaded from the Genomic Data Commons (GDC) TCGA-BRCA project on 10/28/2019^45^. To expedite processing, BAM files were first filtered for all reads aligned to chromosome 5 then this subset was converted to FASTQ format using SamTools v1.9^46^. Reads were aligned to the GRCh38 version of the human transcriptome using STAR v2.7.3a^47^. The Salmon v0.12.0 “quant” mode was used to obtain read counts and TPM measures for each transcript^48^. Patients with expression of the target transcripts hPRLrL (ENST00000618457.5) and hPRLrI (ENST00000619676.4) were verified visually using Integrated Genome Browser based on read alignment covering or spanning, respectively, the documented differential splice junction (corresponding to bp 1010–1581 of the hPRLrL ORF).

### Global differentially-expressed genes analysis

The pre-processed RNAseq HT-Seq-count gene expression values were downloaded from the GDC TCGA-BRCA project on 2/26/2020. Cases were grouped by their ratio of hPRLrI:hPRLrL expression, stratifying by tertile, whereby the top (*n* = 67) and bottom (*n* = 67) tertiles were compared to obtain relevant differentially expressed genes (DEGs). Values were imported into R v3.6.0 for analysis with DESeq2^49,50^. Genes with a total read count across all conditions of 10 or less were removed prior to analysis due to low expression. DESeq2 results were filtered by removing genes with an adjusted p value (padj) ≥ 0.01, an average normalized read count across all conditions (baseMean) ≤10, and a log2 fold change ≥ 1.5 or ≤ −1.5. DEG signatures were determined via Gene Set Enrichment Analysis (GSEA; Broad Institute)^51^.

### Reporting summary

Further information on research design is available in the Nature Research Reporting Summary linked to this article.

## Supplementary information

Supplementary Information

Reporting Summary Checklist

## Data Availability

The data generated and analyzed during this study are described in the following data record: 10.6084/m9.figshare.14135531^52^. All data files are openly available with the data record. A list of file names and the manuscript elements they underlie is also available in the Excel spreadsheet ‘Grible_et_al_underlying_data_list.xlsx’.

## Code availability

All relevant scripts can be found on GitHub at: https://github.com/AmyOlex/hPRLrI_TCGA.
