# Supplementary material for: The human intermediate prolactin receptor is a mammary proto-oncogene
Source: NPJ Breast Cancer. 2021 Mar 26;7:37. doi: 10.1038/s41523-021-00243-7 (PMC7997966; doi:10.1038/s41523-021-00243-7)

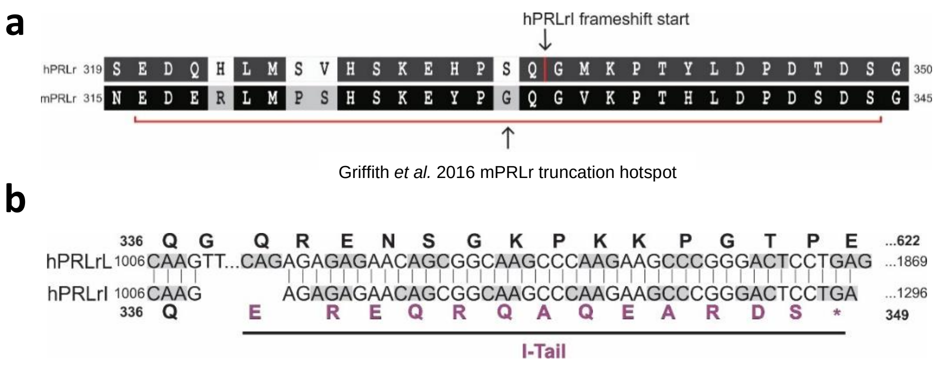

Supplementary Figure 1. hPRLrL, hPRLrI, and mPRLr peptide and nucleotide alignments. a. hPRLr and mPRLr peptide alignment, depicting the mPRLr truncation mutation hotspot described in Griffith *et al.* 2016. **b.** hPRLrL and hPRLrI peptide and nucleotide alignment, highlighting both the region of differential splicing as well as the novel hPRLrI I-Tail. Adapted from Kline *et al.* 1999.

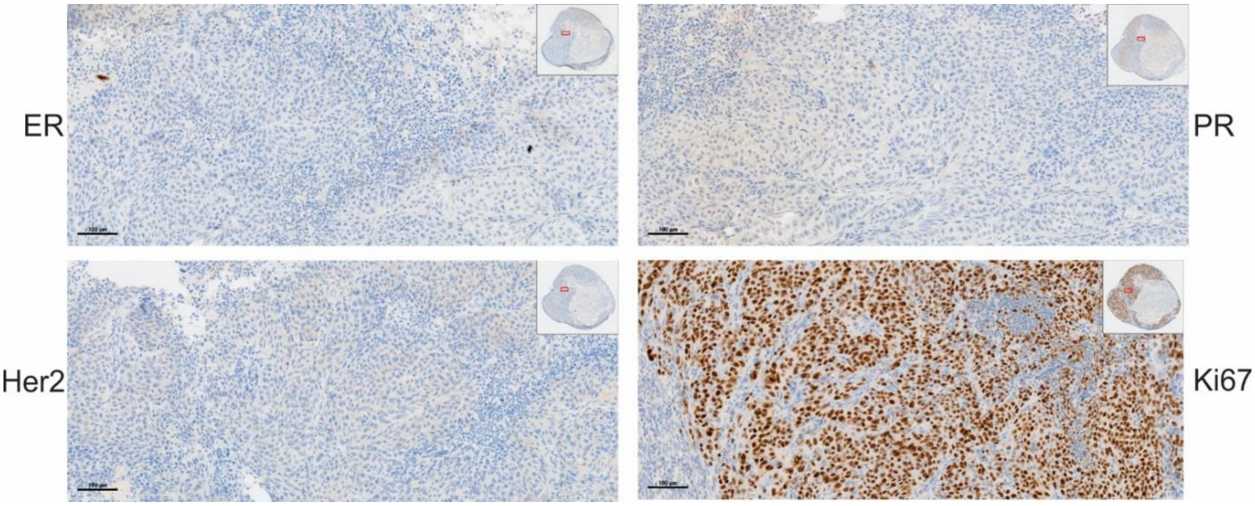

Supplementary Figure 2. hPRLrL+I MCF10AT xenografts form ER/PR/Her2- primary tumors with high Ki67 staining. Hormone receptor and Ki67 status were assessed by IHC. Scale bar: 100µm.

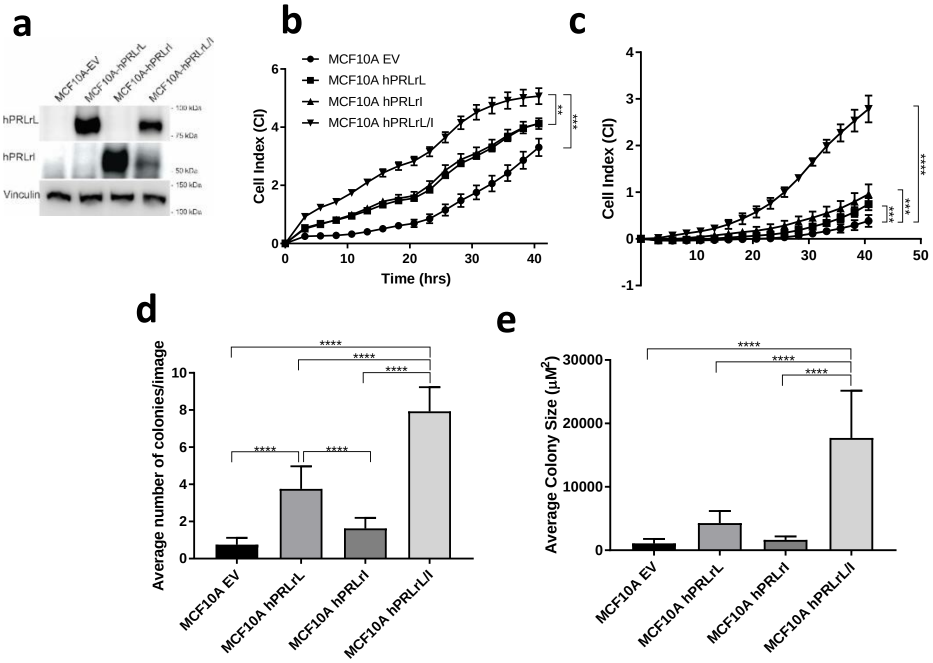

Supplementary Figure 3. MCF10A hPRLrL+I overexpression is transforming *in vitro.* a. MCF10A cells were stably transfected with empty vector, hPRLrL, hPRLrI, or both isoforms together, and respective isoform expression was confirmed via IB. Transfectants were assayed for the differential ability to **b.** proliferate, **c.** migrate, and **d, e** grow in soft agar. **$p < 0.01$, ***$p < 0.005$, ****$p < 0.001$, n=3. Error bars depict SEM.

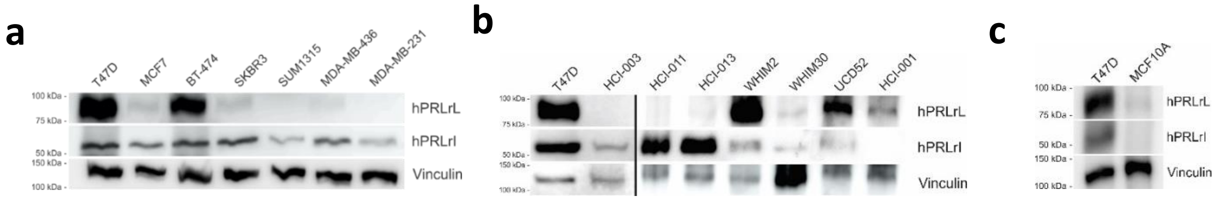

Supplementary Figure 4. hPRLrI is overexpressed in breast cancer. Protein samples from a panel of **a.** breast cancer cell lines, and **b.** patient-derived xenografts (PDX), as well as **c.** normal breast cell line MCF10A were probed for hPRLrL and hPRLrI protein expression via IB. Respective hPRLr isoform identification was determined by assessing molecular mass (hPRLrL: 90kDa; hPRLrI: 55kDa), as compared to T47D positive control cells.

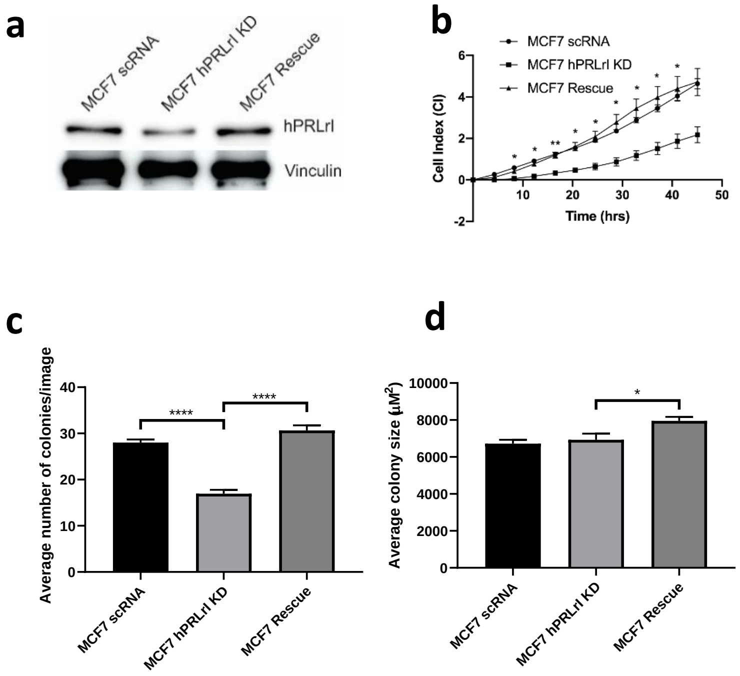

Supplementary Figure 5. hPRLrI overexpression rescue in MCF7 KD cells is sufficient to rescue the malignant phenotype *in vitro.* MCF7 hPRLrI KD cells were stably transfected with hPRLrI cDNA, and **a.** protein expression was evaluated by IB. **b.** Proliferation was assessed using an xCELLigence apparatus, and rescue of anchorage-independent colony formation was assessed by soft agar, quantifying both **c.** colony number and **d.** colony size. *$p < 0.05$, **$p < 0.01$, ****$p < 0.001$. n=3. Error bars depict SEM.

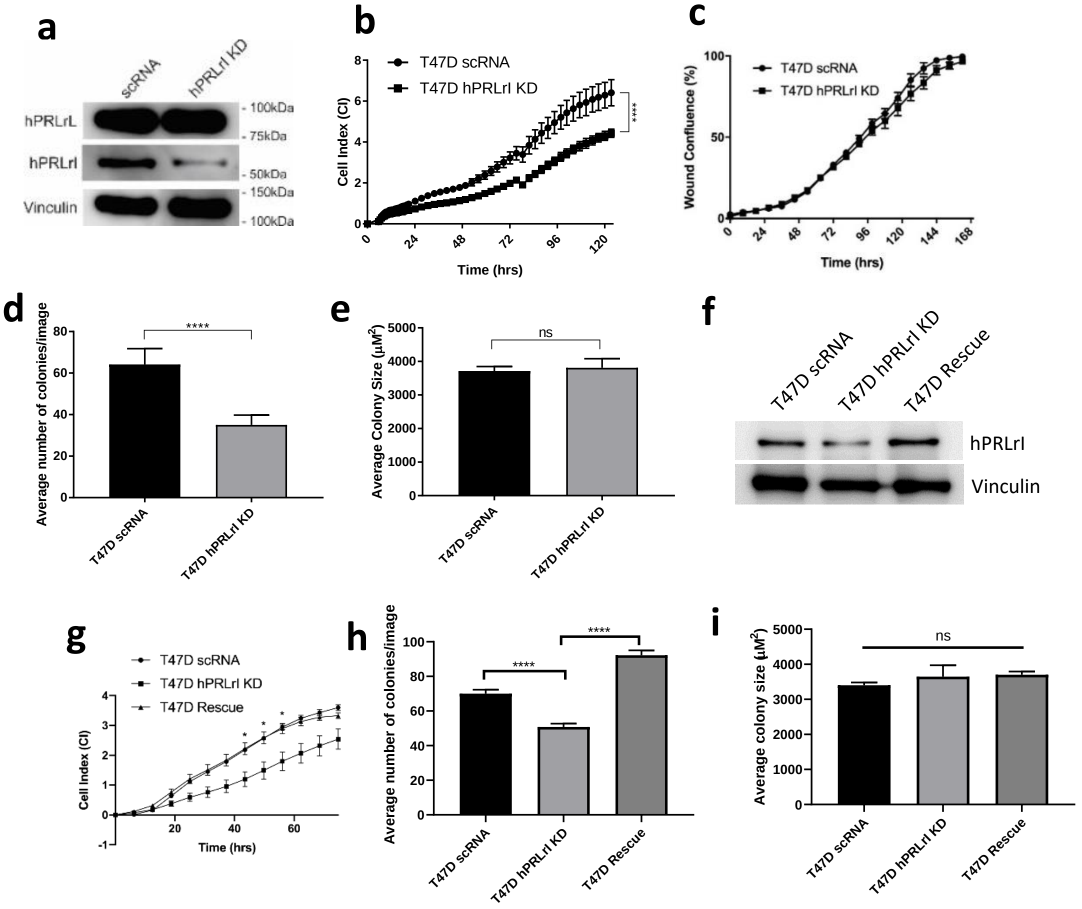

Supplementary Figure 6. hPRLrI KD in T47D cells reduces transforming potential *in vitro*, which is subsequently rescued by hPRLrI overexpression. a. T47D cells were stably transfected with anti-hPRLrI shRNA, and KD efficiency was assessed by IB. **b.** Differential proliferation, **c.** migration, and **d, e** colony formation in soft agar were assessed. **f.** Stable hPRLrI over-expression rescue was confirmed by IB, and cells were assessed for their rescue of **g.** proliferative potential as well as **h, i** growth in soft agar. $*p < 0.05$, $****p < 0.001$. n=3. Error bars depict SEM.

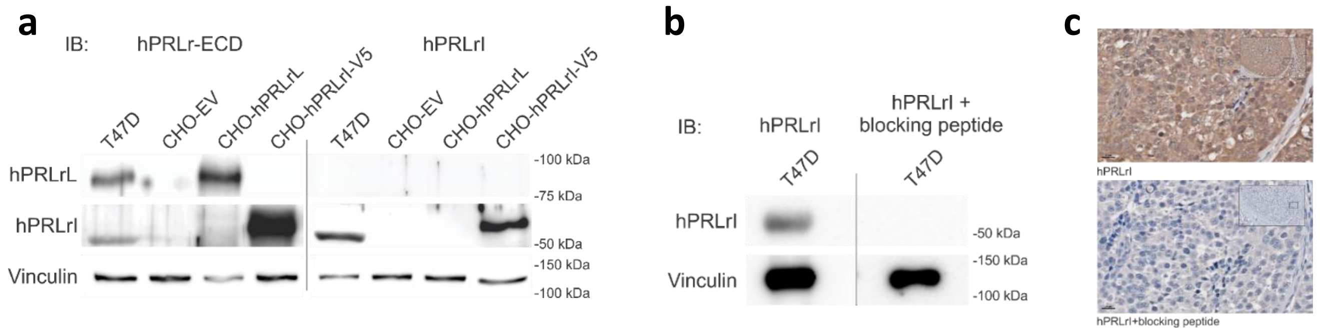

Supplementary Figure 7. Generation of an hPRLrI-specific polyclonal antibody. An anti-hPRLrI antibody, recognizing the unique I-Tail, was generated, and specificity was determined through a**.** IB of T47D and CHO transfectant lysates, **b.** IB of T47D lysates in the presence/absence of an hPRLrI antibody blocking peptide, and **c.** IHC of a human breast cancer tissue sample, also in the presence/absence of the aforementioned blocking peptide.

		hPRLrI^{hi}/hPRLrL^{lo} (n = 67)		hPRLrI^{lo}/hPRLrL^{hi} (n = 67)			
		**n**	**percent of total**	**n**	**percent of total**	**z score**	**p**
**ER status**							
	Positive	48	71.6%	55	82.1%	-1.434	0.152
	Negative	11	16.4%	4	6.0%	1.918	0.055
**PR status**							
	Positive	41	61.2%	47	70.1%	-1.092	0.275
	Negative	17	25.4%	11	16.4%	1.275	0.202
**Her2 status**							
	Positive	11	16.4%	10	14.9%	0.238	0.812
	Negative	35	52.2%	36	53.7%	-0.173	0.863
	Equivocal	9	13.4%	11	16.4%	-0.485	0.628
**HR status**							
	ER+/PR+/Her2-	25	37.3%	31	46.3%	-1.051	0.293
	ER+/PR-/Her2-	5	7.5%	3	4.5%	0.729	0.466
	ER-/PR-/Her2+	2	3.0%	2	3.0%	0.000	1.000
	ER-/PR-/Her2-	4	6.0%	1	1.5%	1.367	0.172
**Intrinsic subtype**							
	Luminal A	34	50.7%	44	65.7%	-1.752	0.080
	Luminal B	14	20.9%	12	17.9%	0.437	0.662
	Her2-enriched	2	3.0%	5	7.5%	-1.165	0.244
	Basal	11	16.4%	1	1.5%	3.025	**<u>0.002</u>**
	Normal-like	1	1.5%	0	0.0%	1.004	0.315
**Gender**							
	Female	64	95.5%	66	98.5%	-1.015	0.310
	Male	3	4.5%	1	1.5%	1.015	0.310
**Menopause status**							
	Pre-menopausal	16	25.0%	15	22.7%	0.304	0.761
	Peri-menopausal	1	1.6%	1	1.5%	0.022	0.983
	Post-menopausal	38	59.4%	44	66.7%	-0.861	0.389
**Race**							
	White	55	82.1%	58	86.6%	-0.713	0.476
	Asian	1	1.5%	2	3.0%	-0.584	0.559
	African American	4	6.0%	3	4.5%	0.388	0.698
**Ethnicity**							
	Not hispanic or latino	51	76.1%	58	86.6%	-1.552	0.121
	Hispanic or latino	3	4.5%	2	3.0%	0.456	0.649
**Pathologic T**							
	T1	13	19.4%	24	35.8%	-2.125	**<u>0.034</u>**
	T2-T4	54	80.6%	43	64.2%	2.125	**<u>0.034</u>**
**Pathologic N**							
	N0	27	40.3%	36	53.7%	-1.558	0.119
	N1-N3	38	56.7%	31	46.3%	1.210	0.226
**Pathologic M**							
	M0	55	82.1%	61	91.0%	-1.520	0.129
	M1	2	3.0%	2	3.0%	0.000	1.000
**Pathologic Stage**							
	Stage I	8	11.9%	17	25.4%	-1.996	**<u>0.046</u>**
	Stage II-Stage IV	57	85.1%	49	73.1%	1.700	0.089
**Age**							
	<55	26	38.8%	23	34.3%	0.538	0.591
	>55	41	61.2%	44	65.7%	-0.538	0.591
**Prior malignancy**							
	No	61	91.0%	65	97.0%	-1.458	0.145
	Yes	6	9.0%	2	3.0%	1.458	0.145

Supplementary Table 1. TCGA BRCA samples were stratified into tertiles based on ratio of hPRLrI:hPRLrL transcript expression. Clinicopathological features were obtained using the Genomic Data Commons (GDC) Data Portal (portal.gdc.cancer.gov), and significance between cohorts was assessed by z-score and two-tailed p-value. Significant values are bolded and underlined.

Figure 2a/2b

MCF10AT-EV

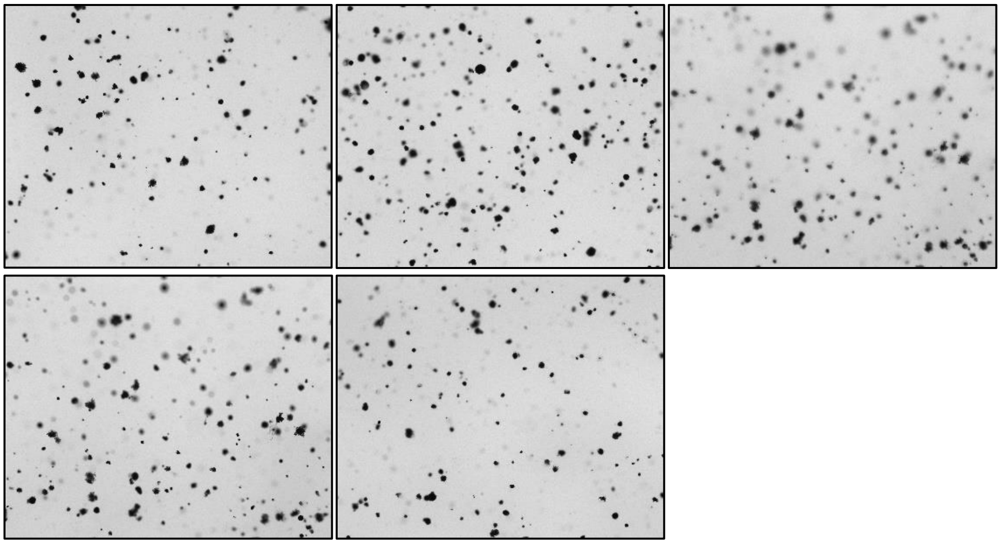

MCF10AT-hPRLrL

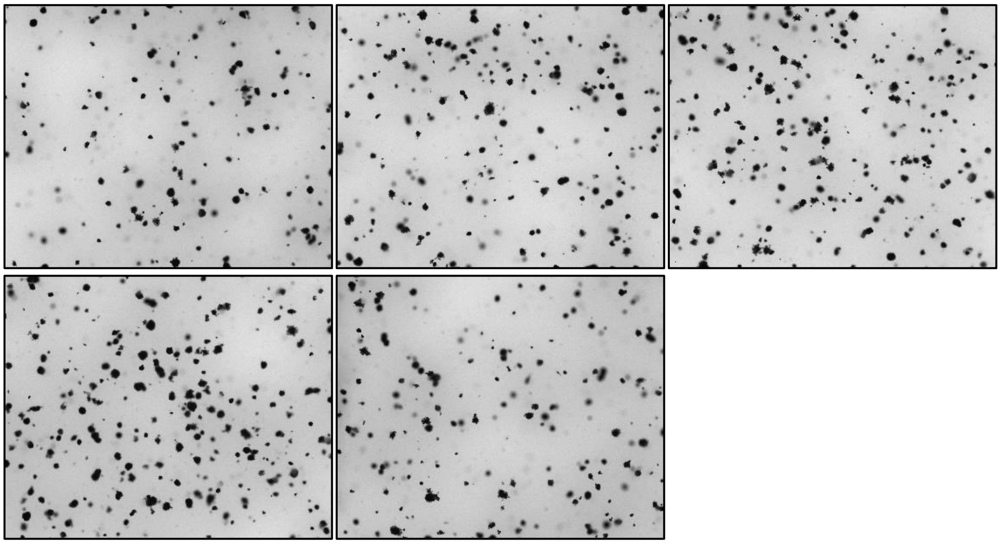

MCF10AT-hPRLrI

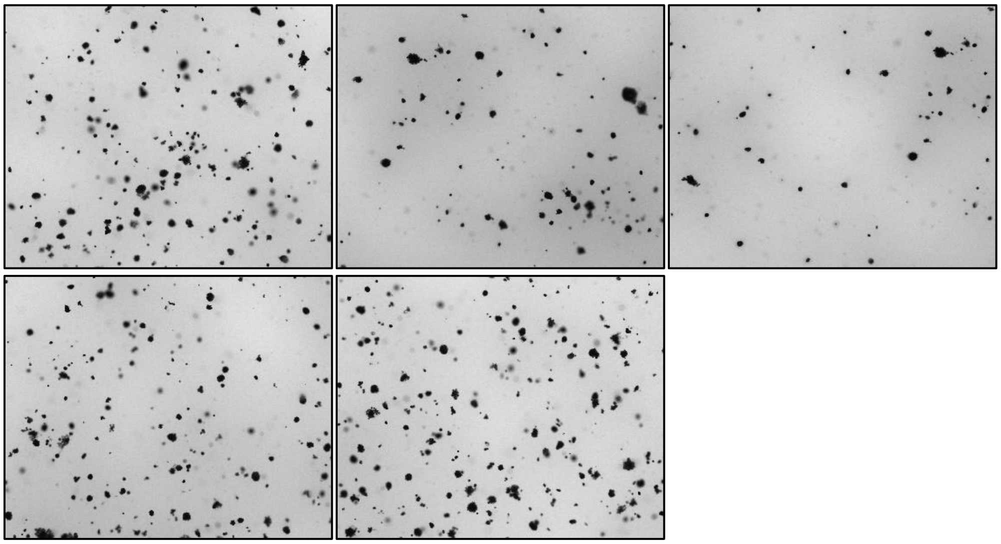

MCF10AT-hPRLrL+I

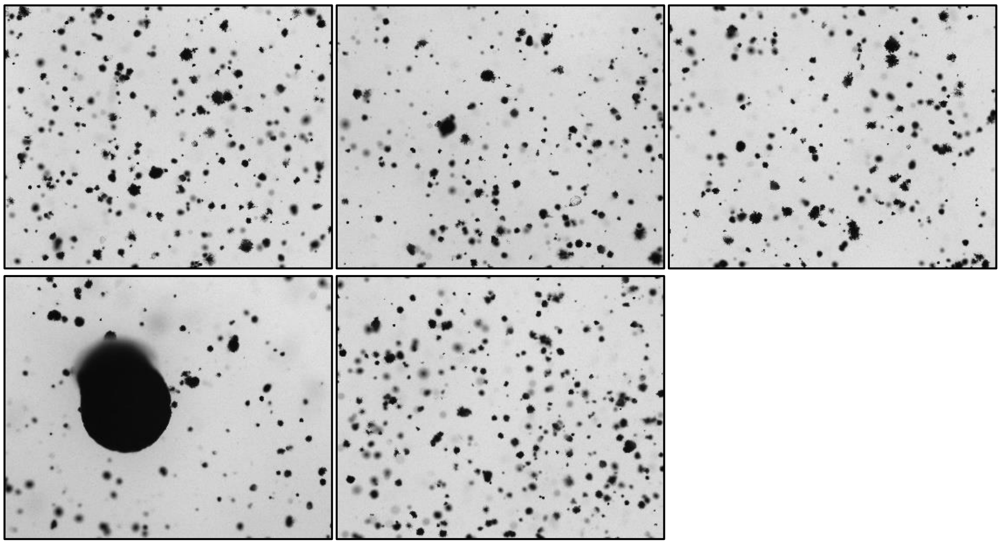

Figure 3b/3c

MCF7 scRNA

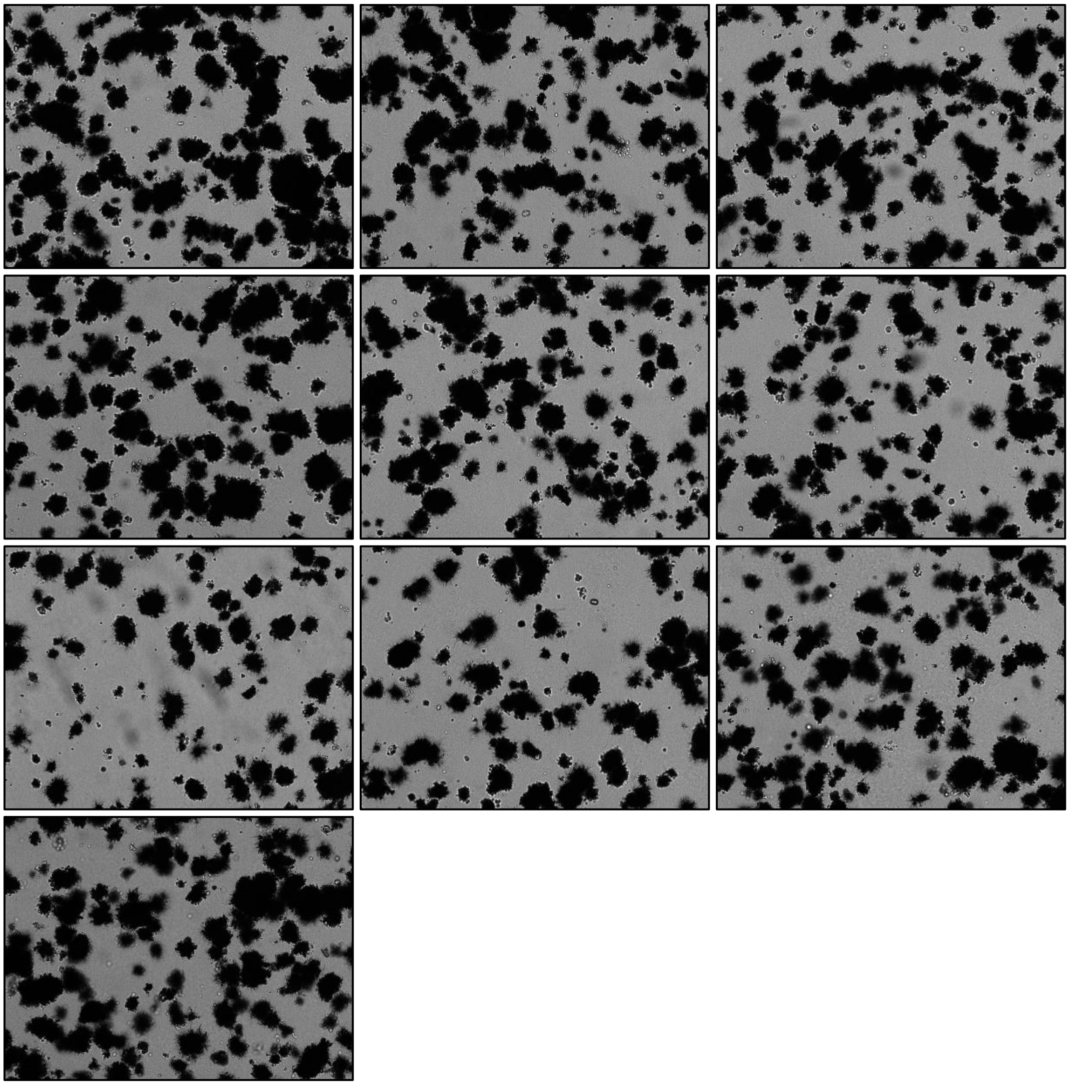

MCF7 hPRLrI KD

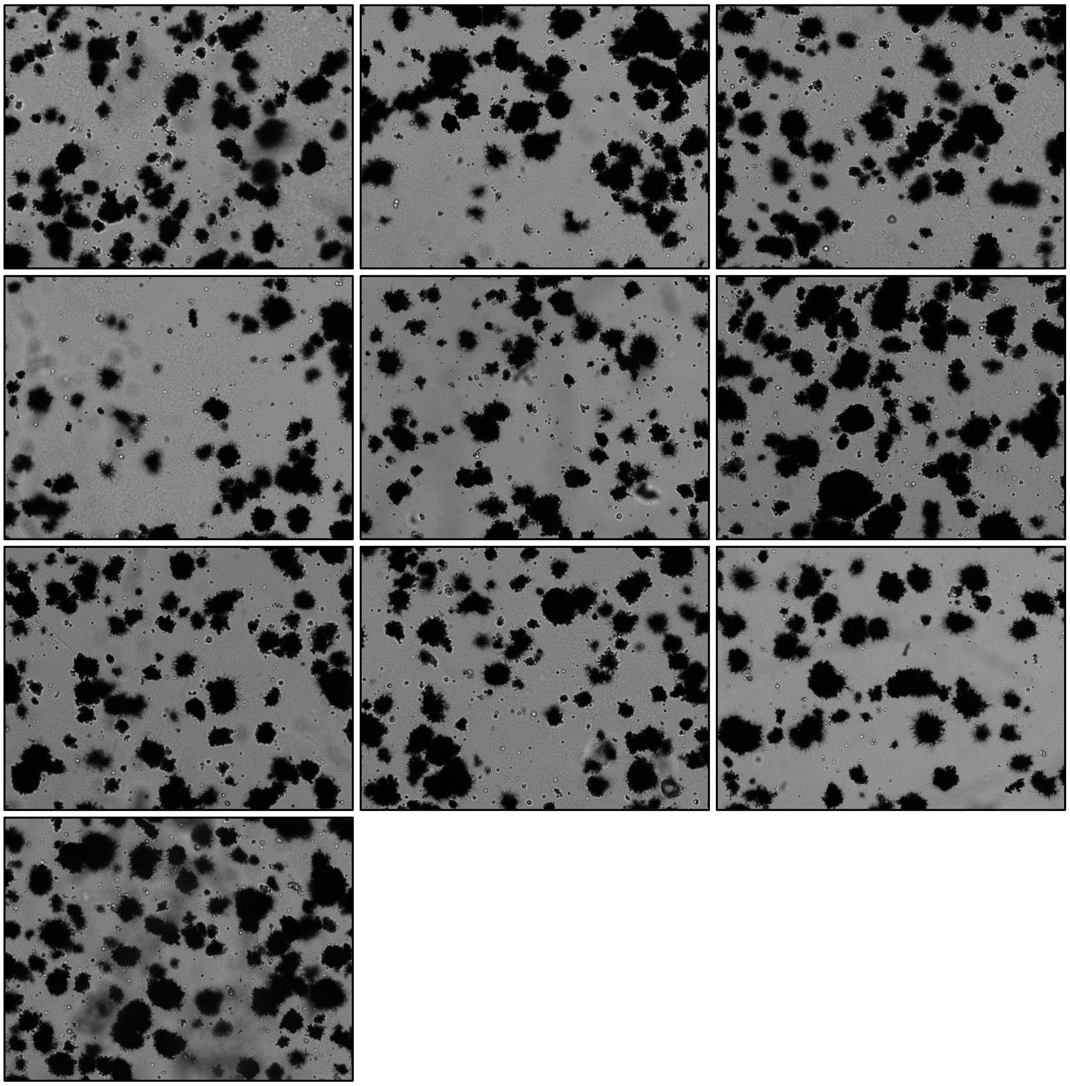

Figure 6b/6c

MCF10AK-EV

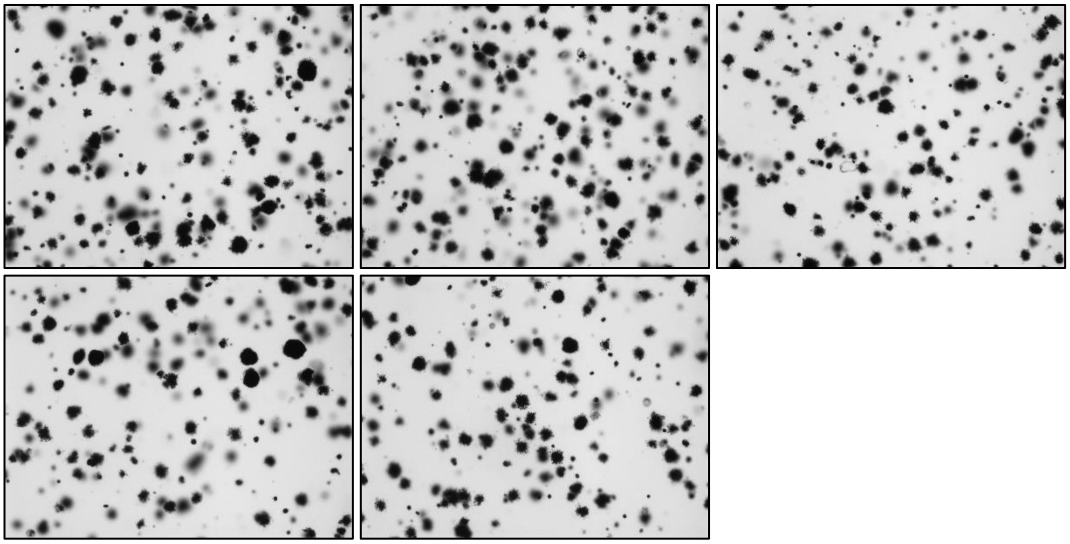

MCF10AK-hPRLrL

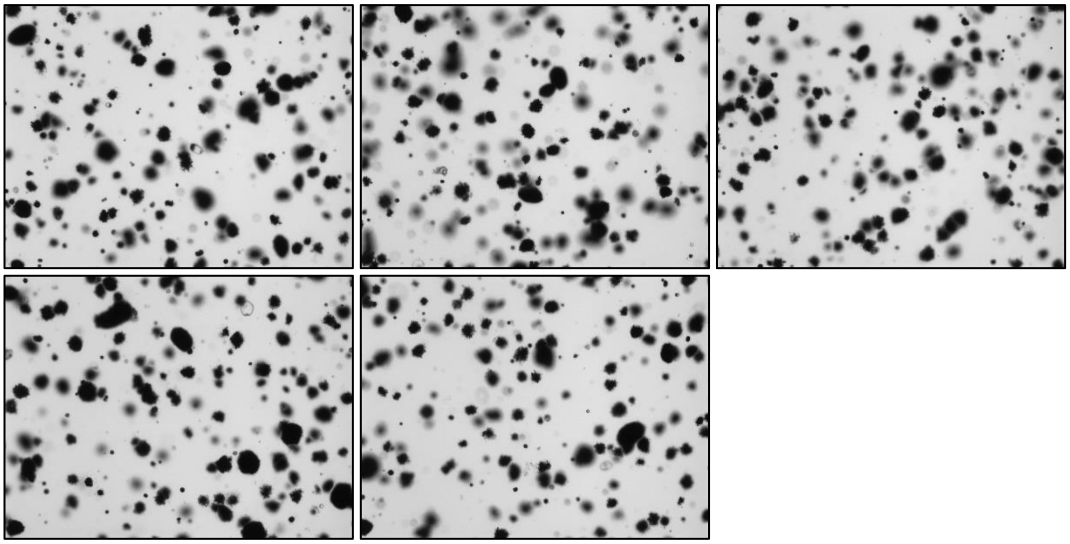

MCF10AK-hPRLrI

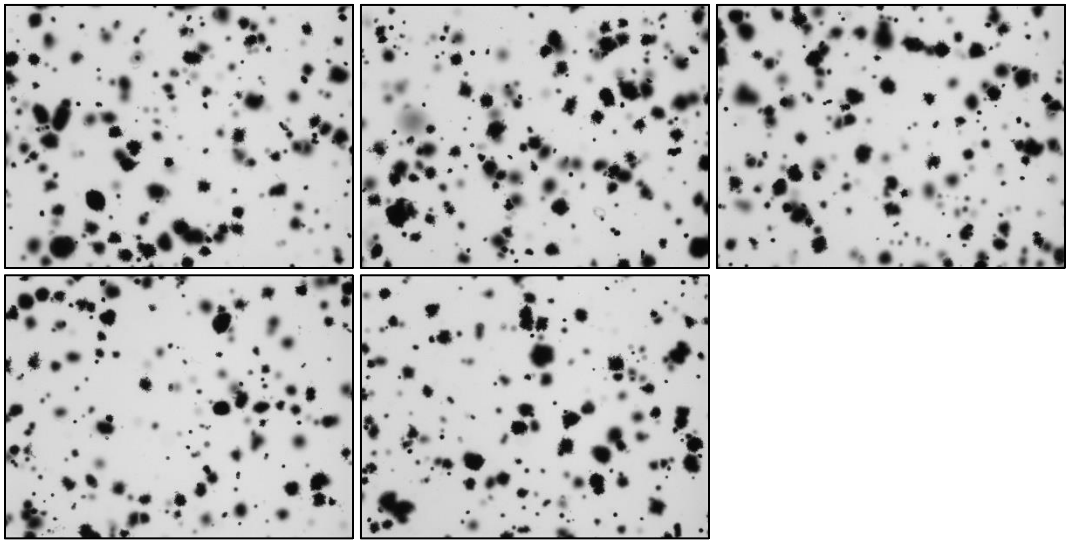

MCF10AK-hPRLrL+I

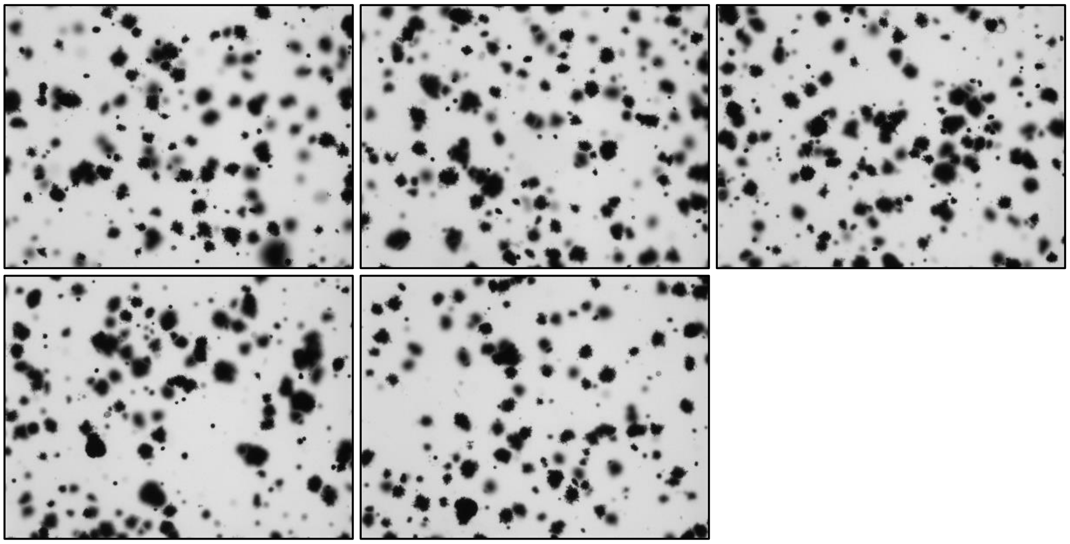

Supplementary Figure 2d/2e

MCF10A-EV

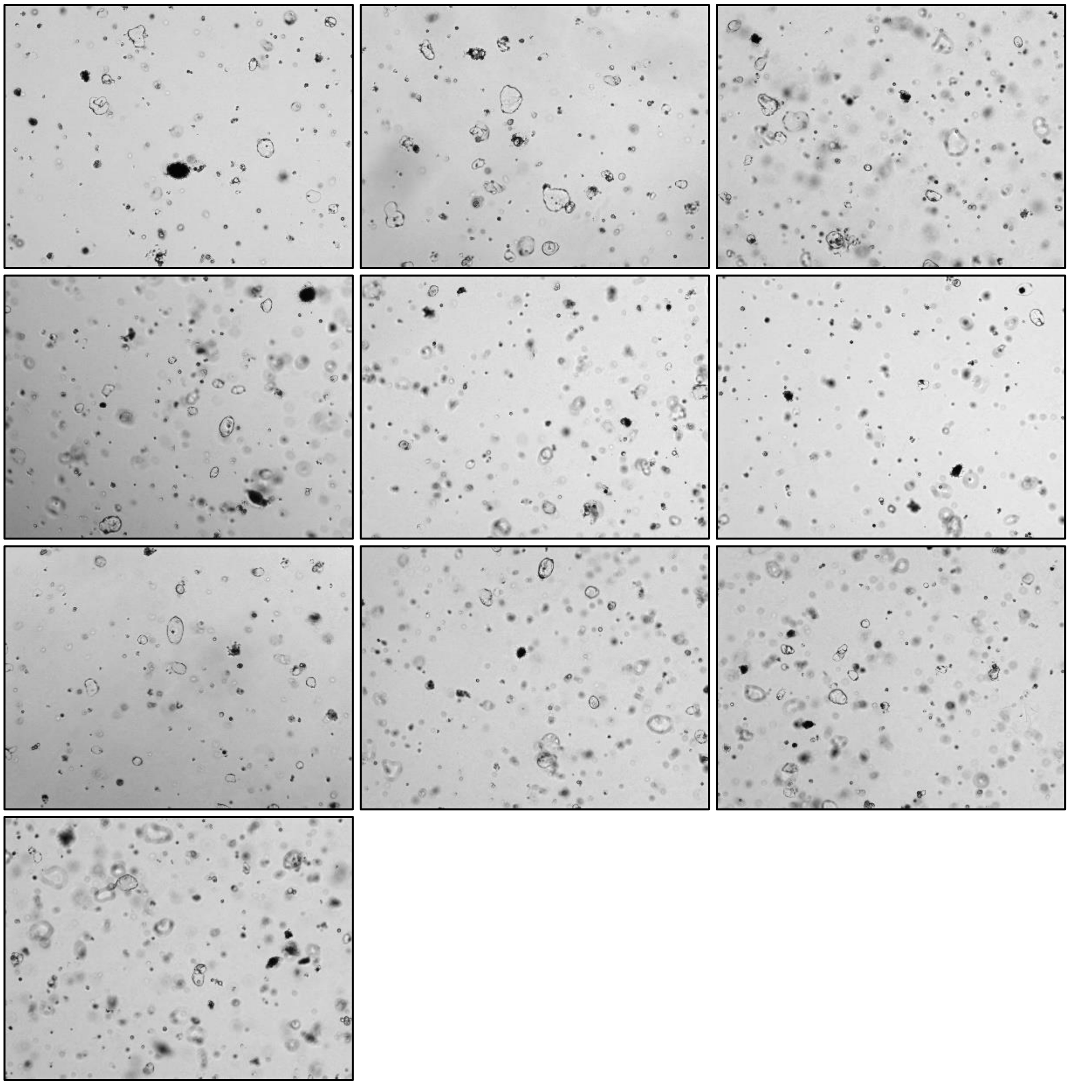

MCF10A-hPRLrL

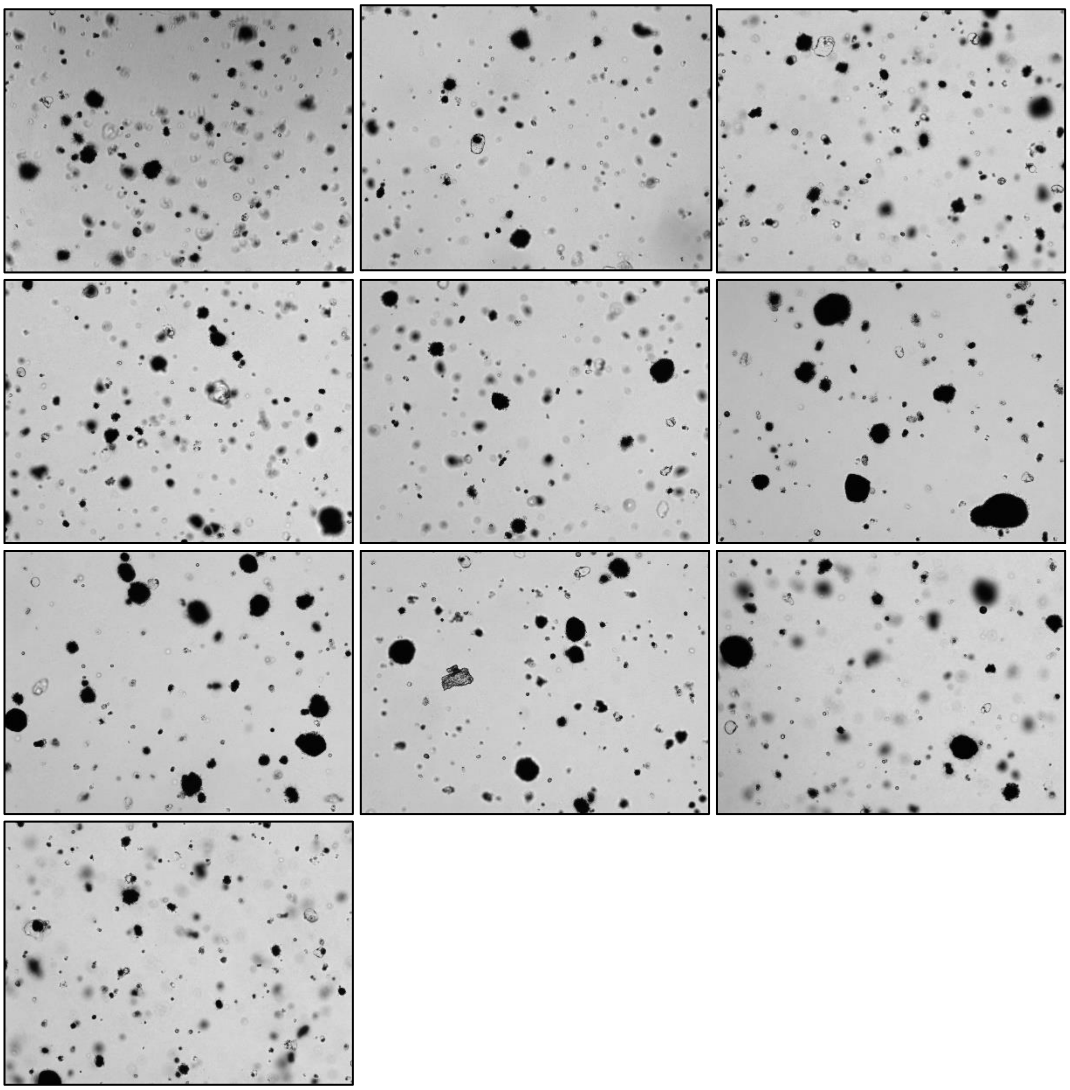

MCF10A-hPRLrI

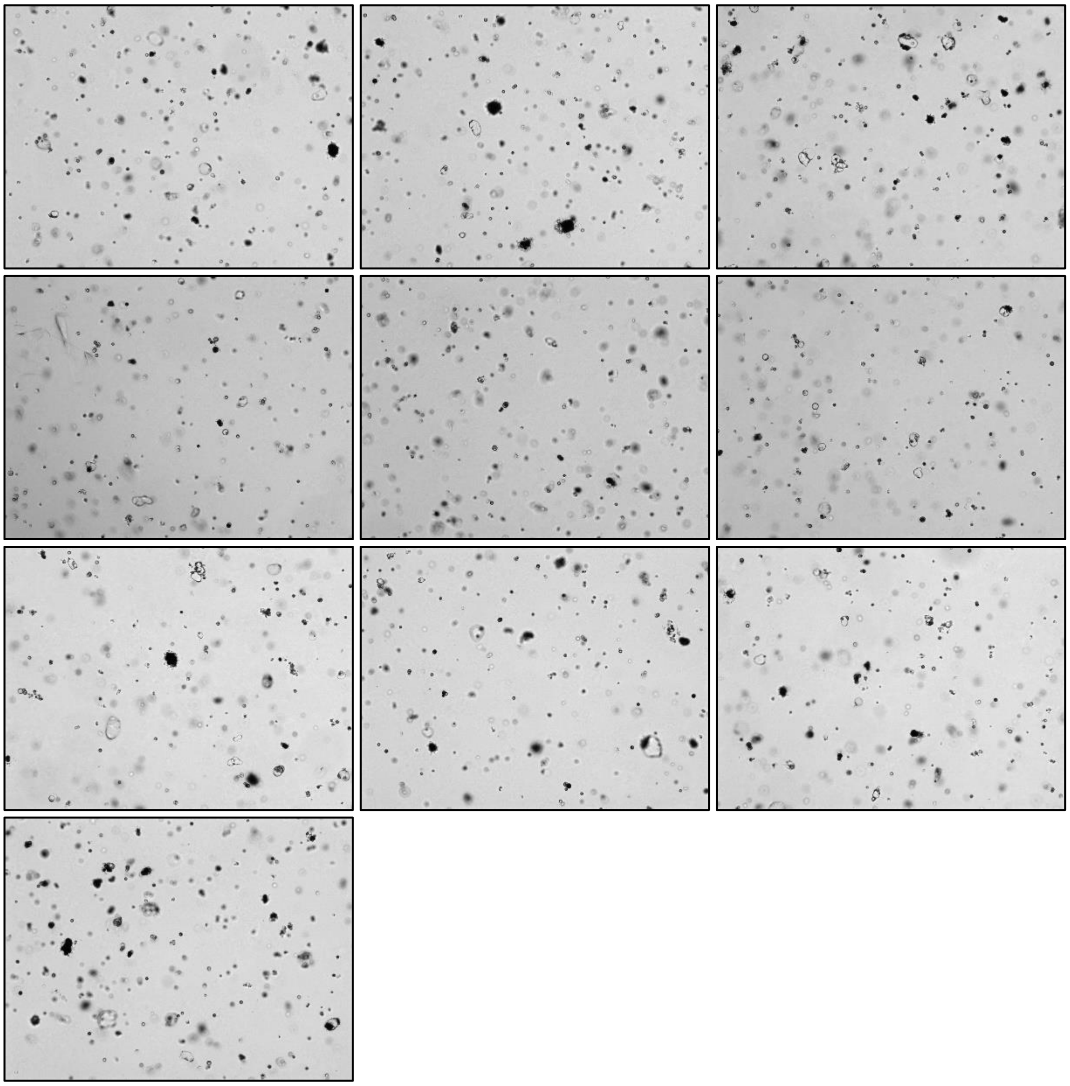

MCF10A-hPRLrL+I

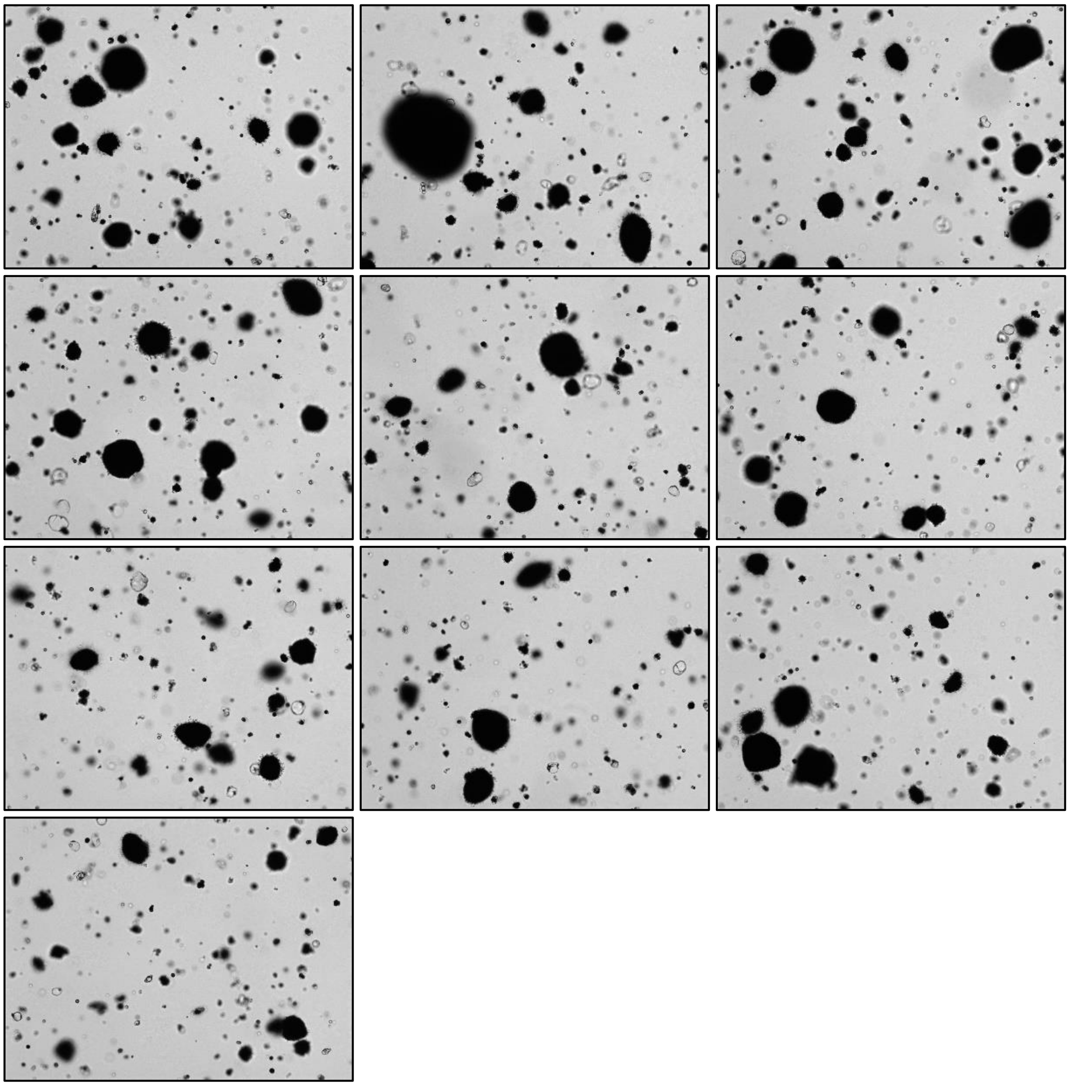

Supplementary Figure 4c/d

MCF7 scRNA

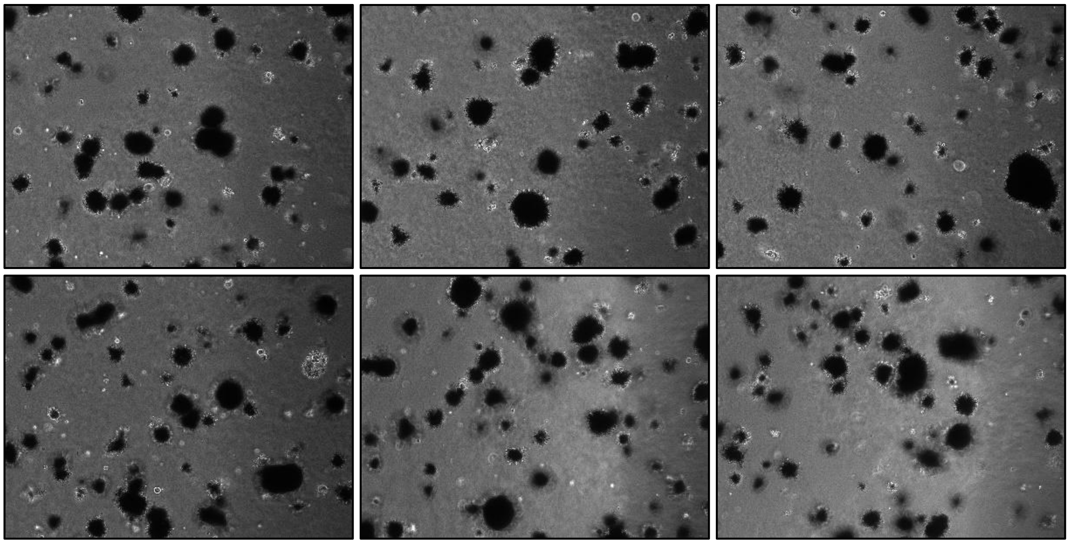

MCF7 hPRLrI KD

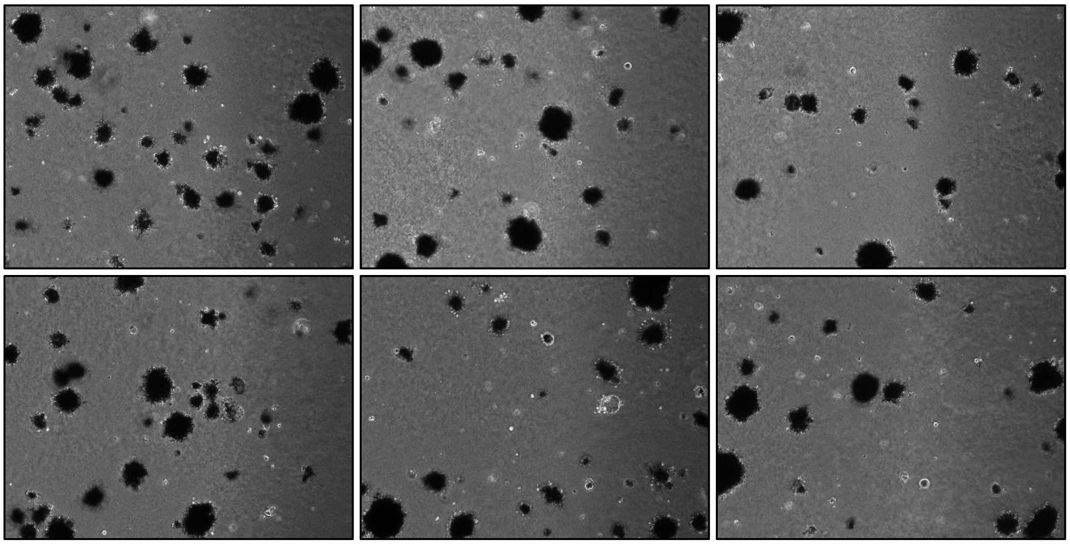

MCF7 Rescue

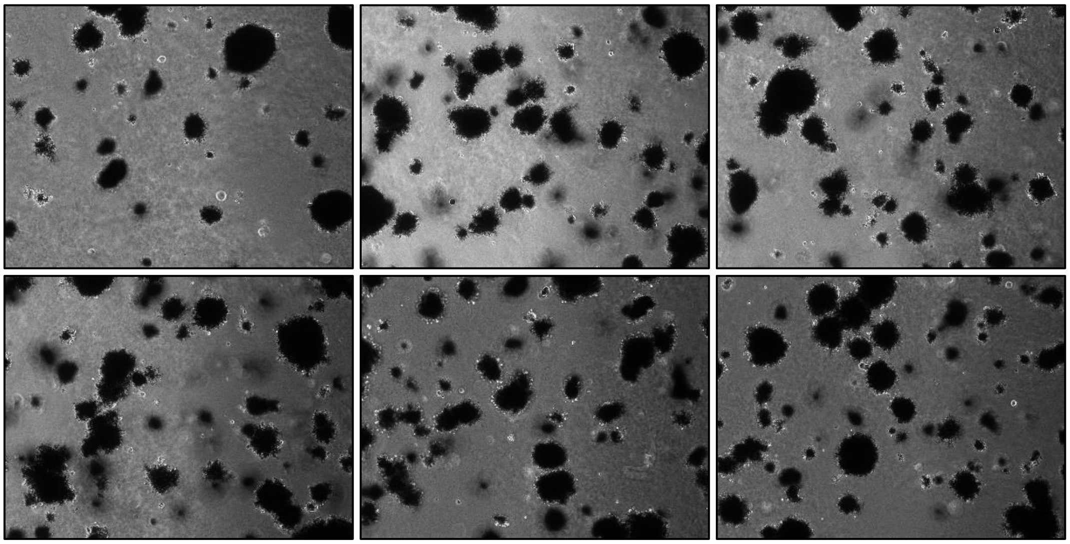

Supplementary Figure 5d/e/h/i

T47D scRNA

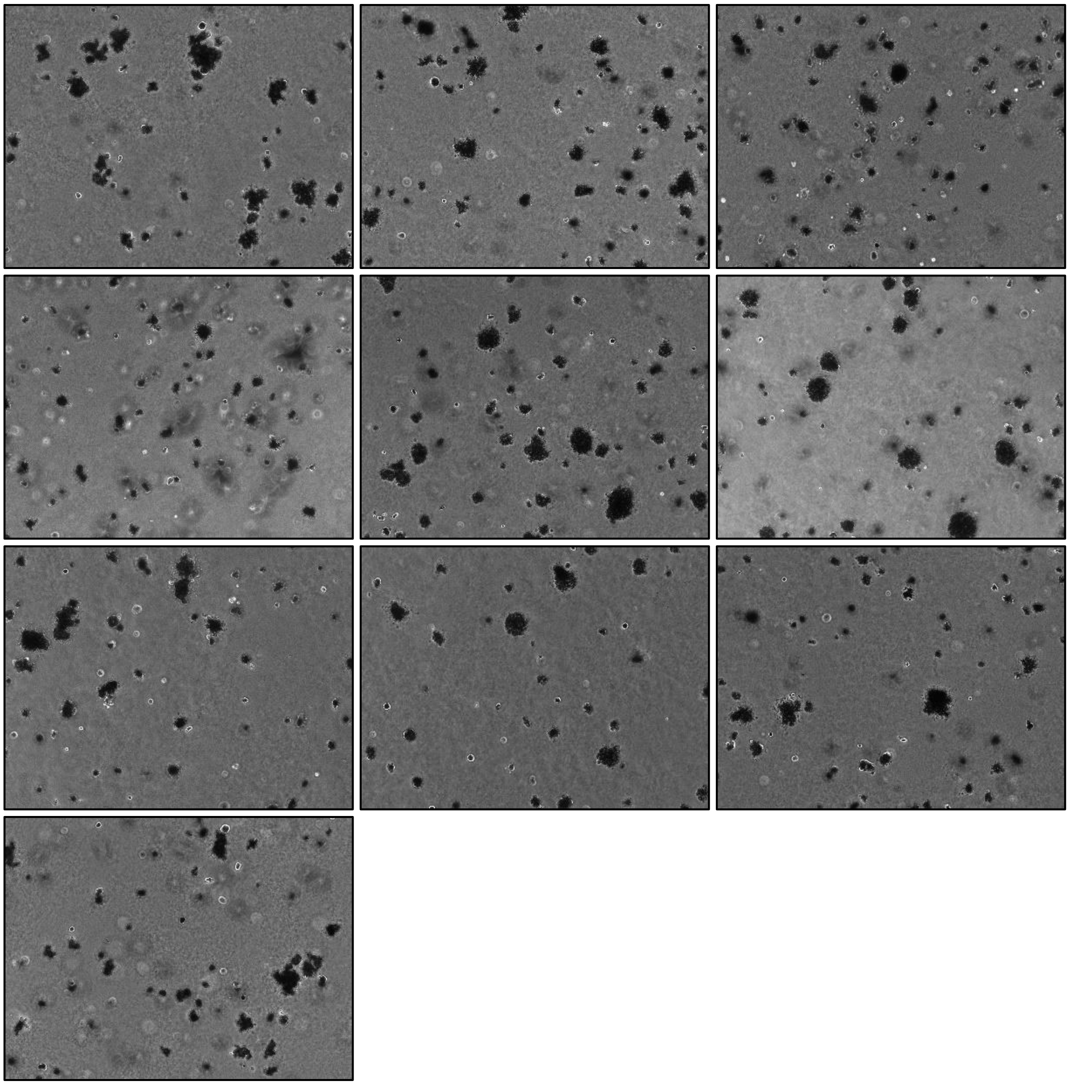

T47D hPRLrI KD

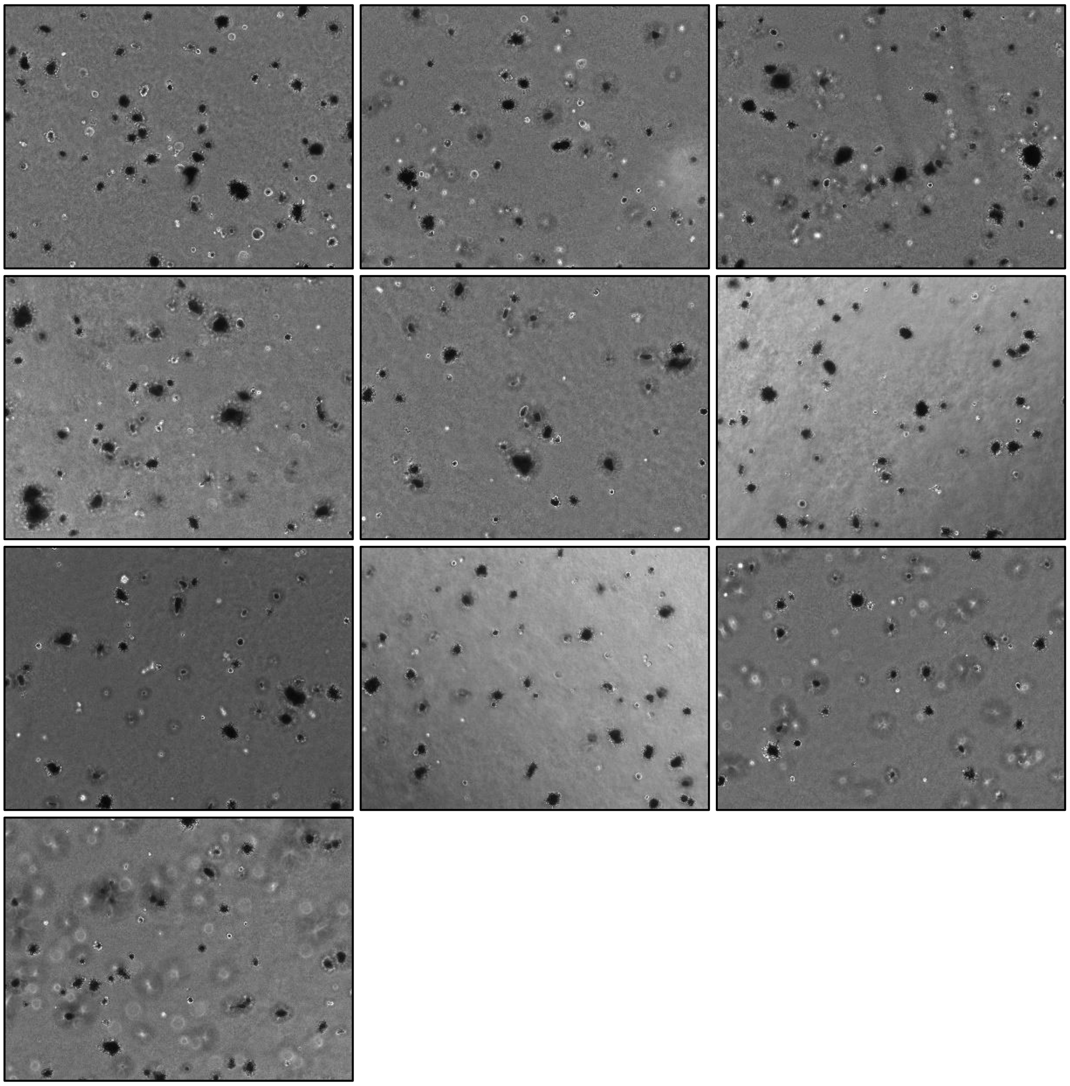

T47D Rescue

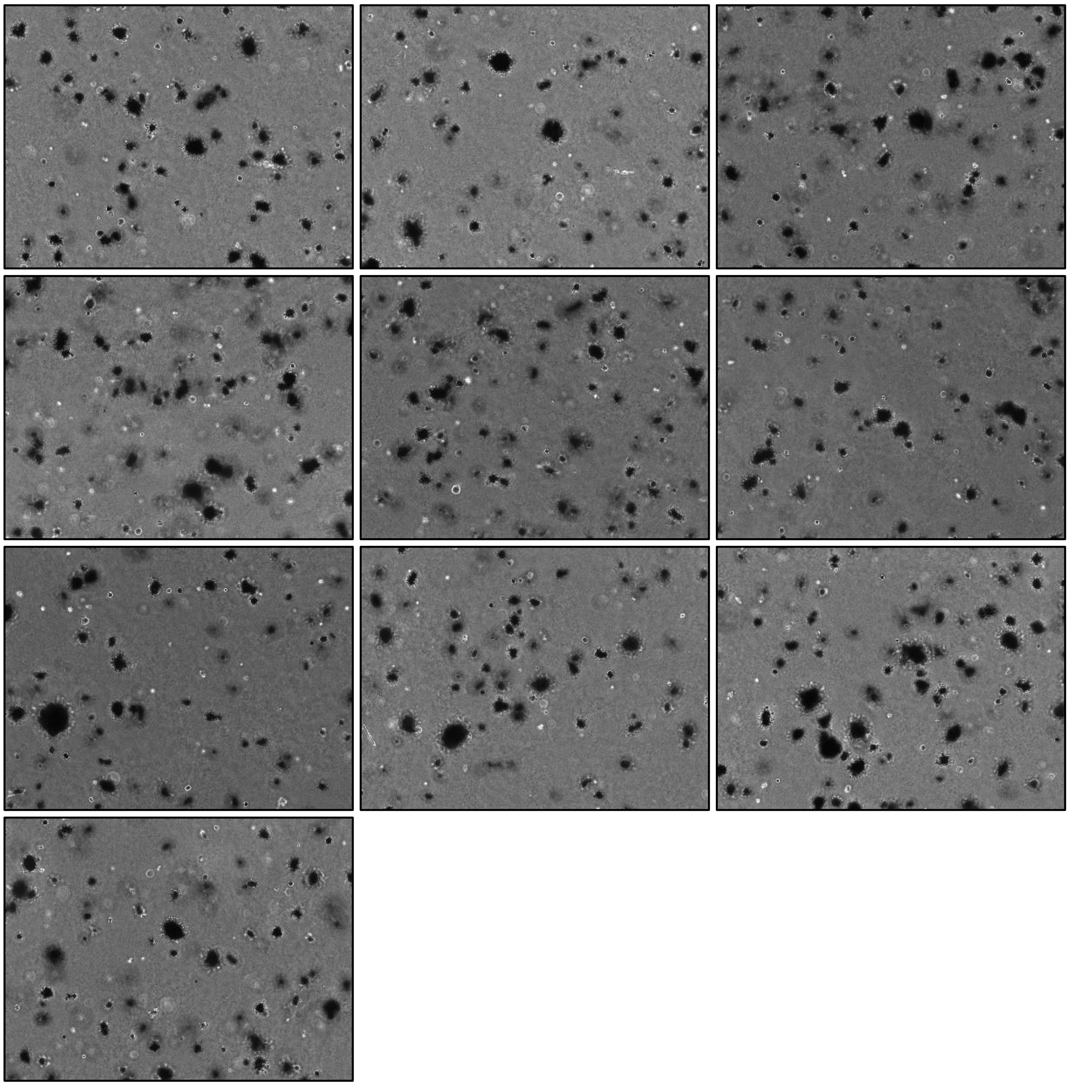

Figure 1A.

IB: hPRLr ECD

IB: Vinculin

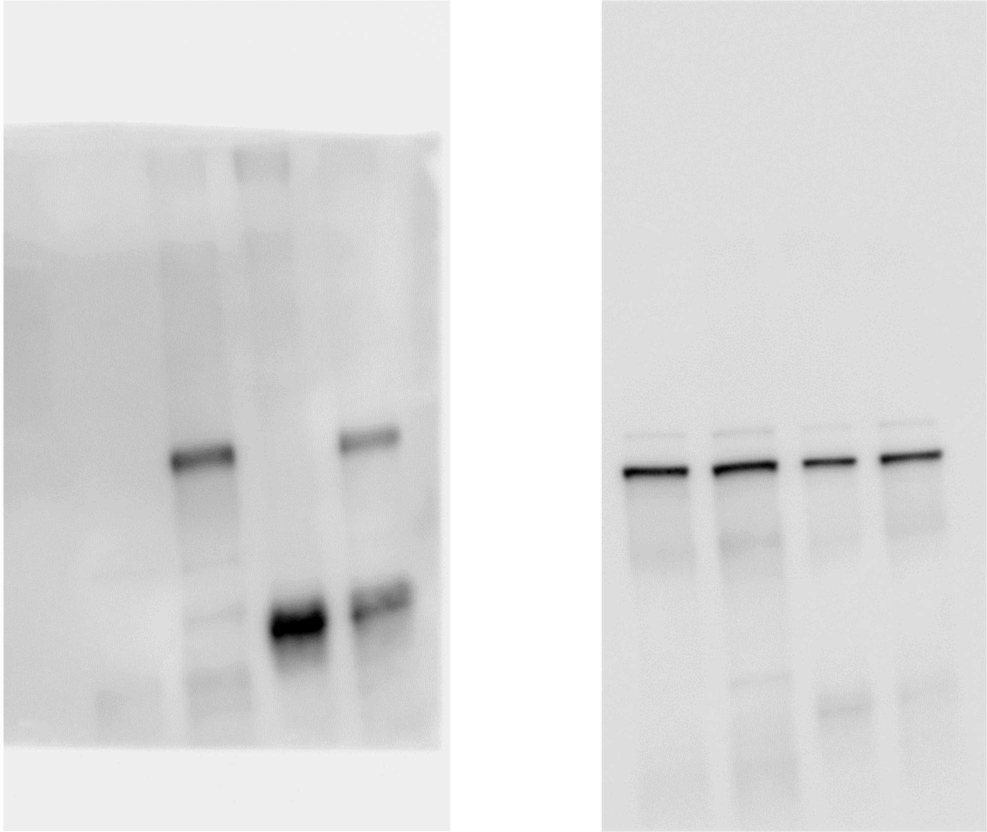

Figure 3A.

IB: hPRLrI **IB: hPRLr-ECD** **IB: Vinculin**

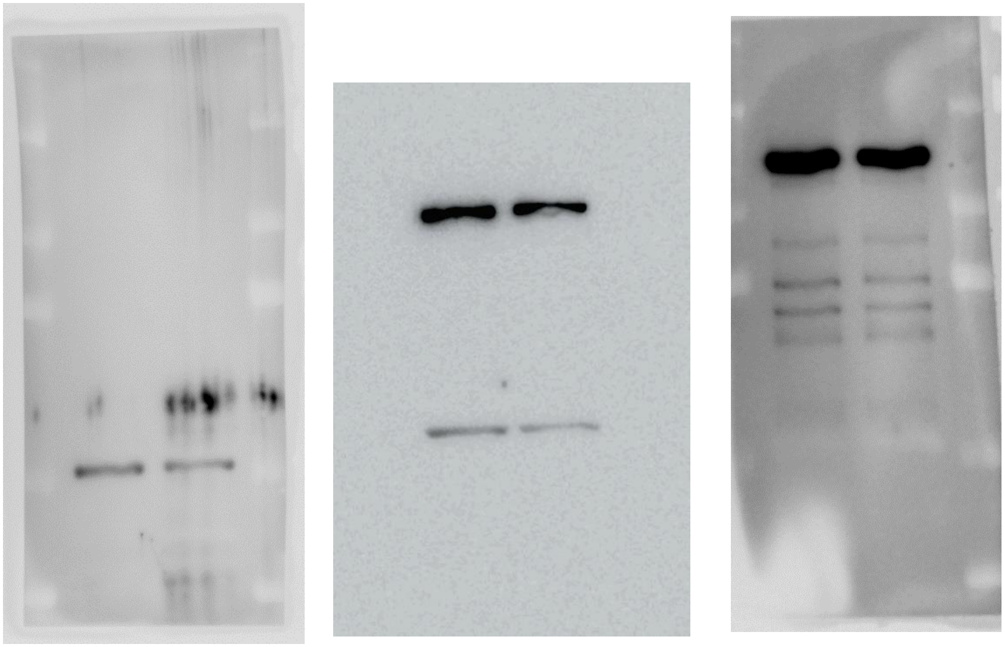

Figure 4A.

IB: hPRLr-ECD

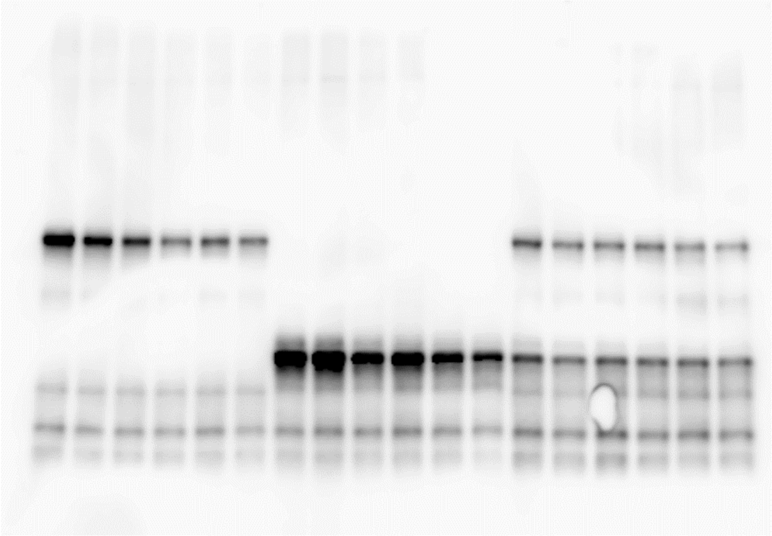

IB: Vinculin

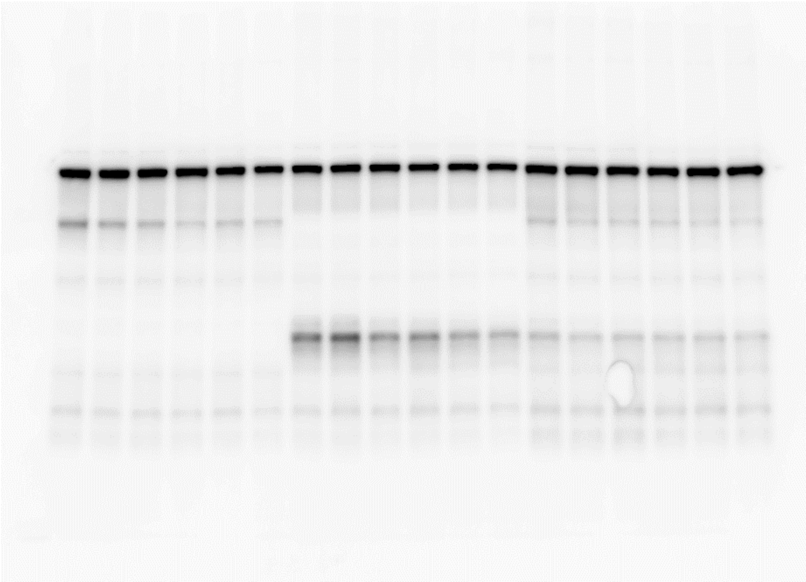

Figure 4C.

IB: pS349-hPRLr

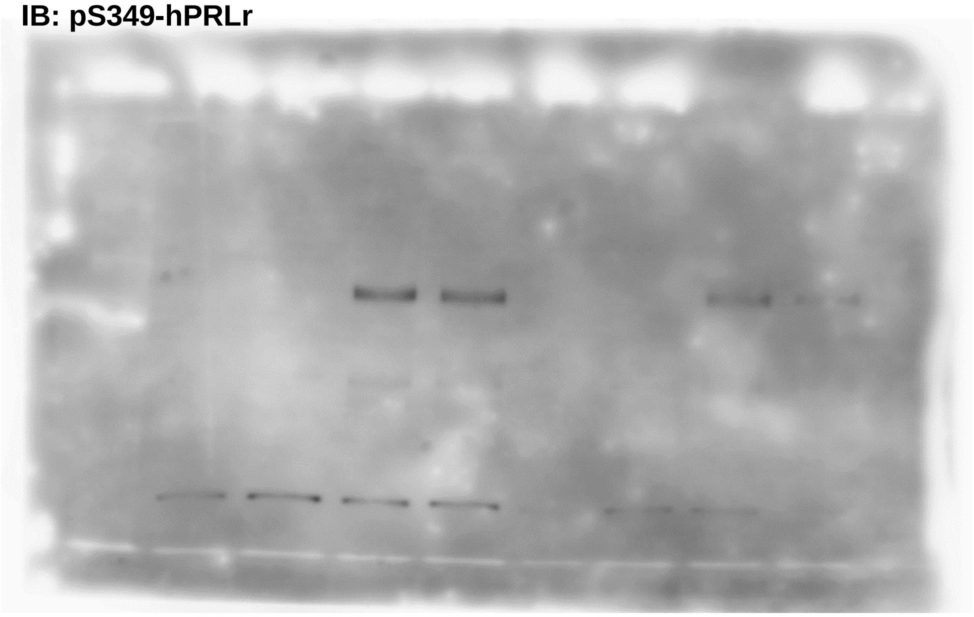

IB: hPRLr-ECD

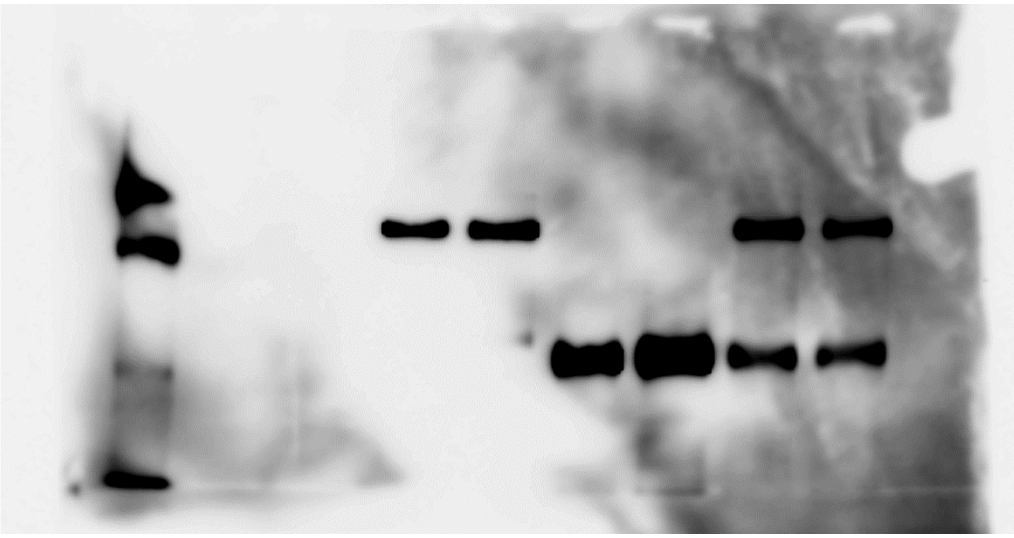

Figure 4C.

IB: Vinculin

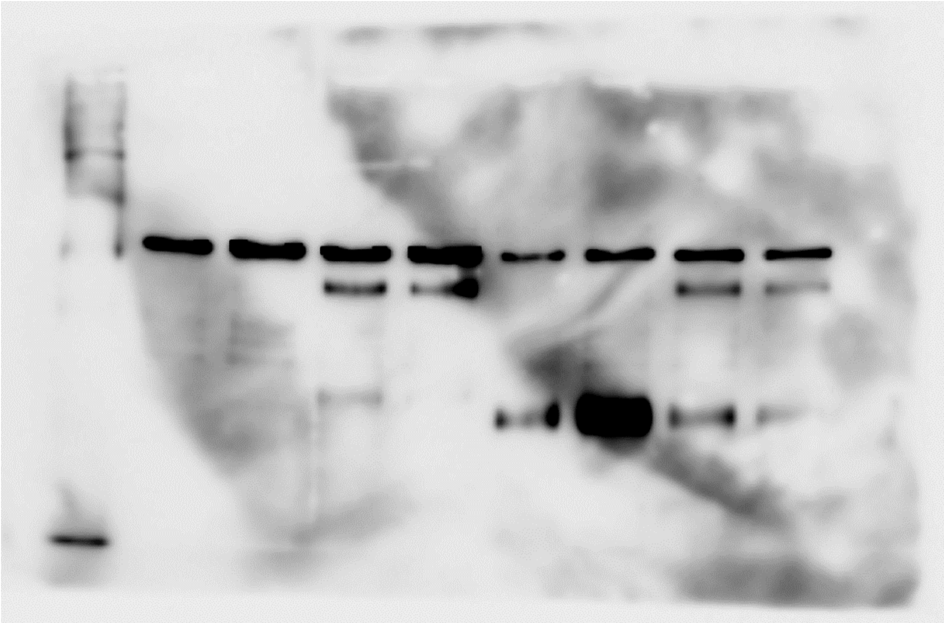

Figure 5A.

IB: pY-Jak2

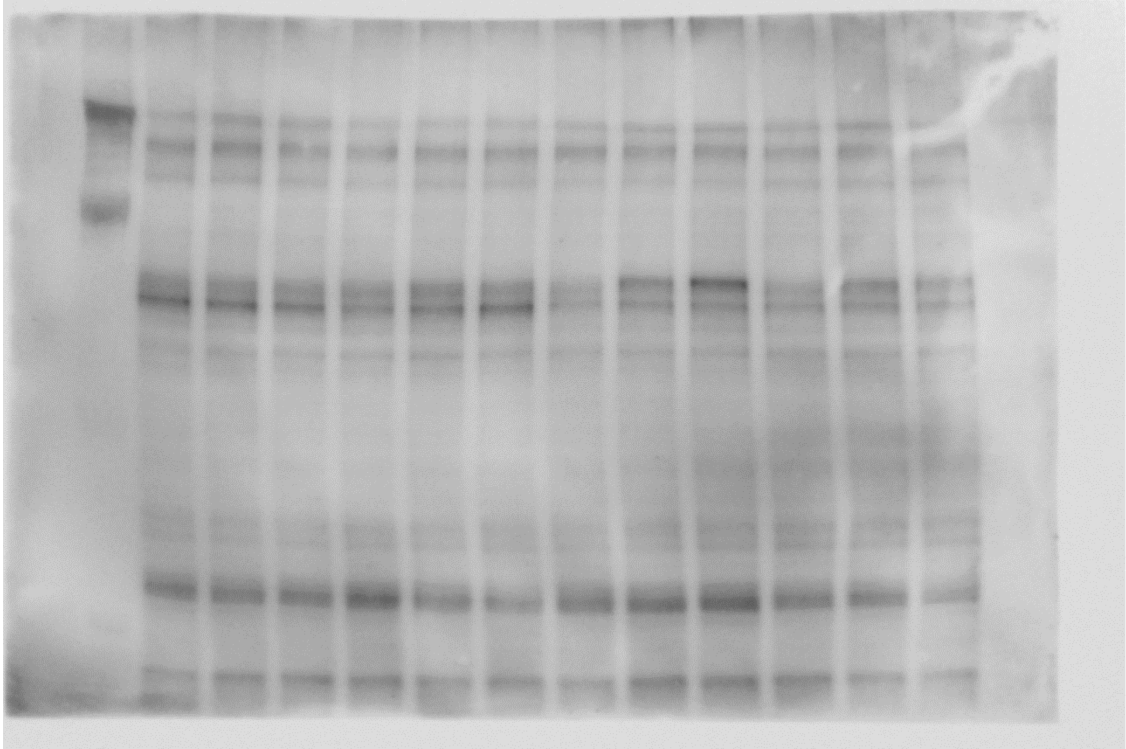

IB: Jak2

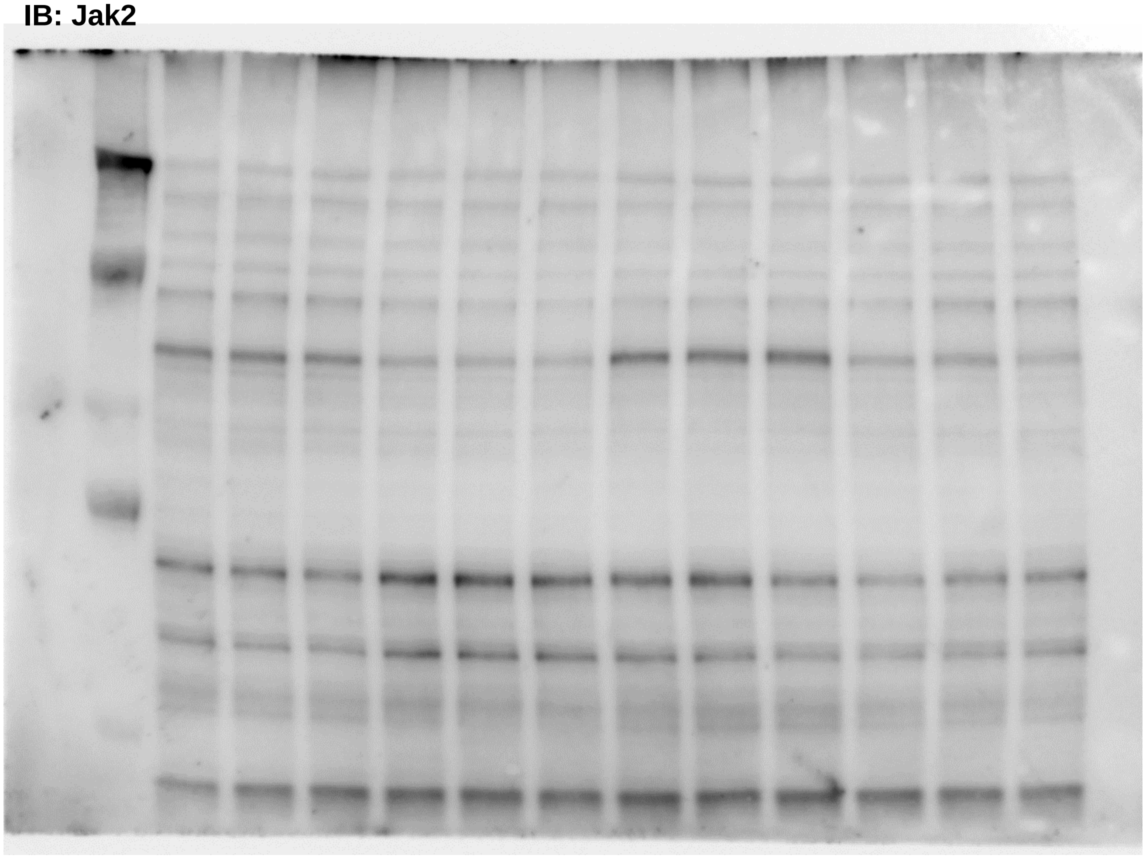

Figure 5A.

IB: pY-Stat5a

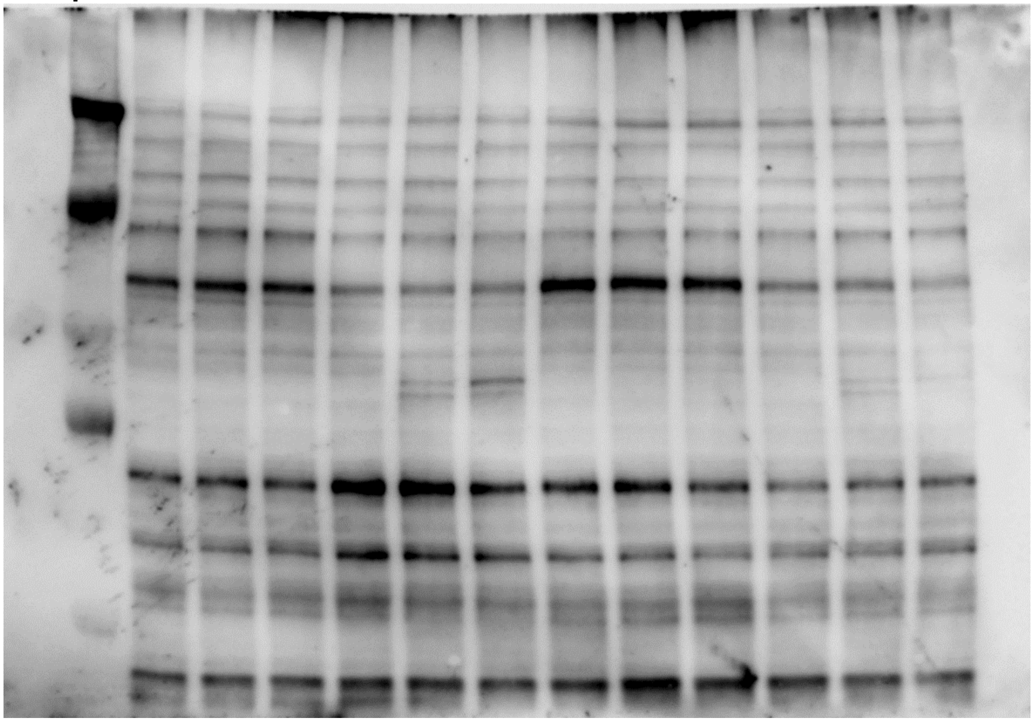

IB: Stat5a

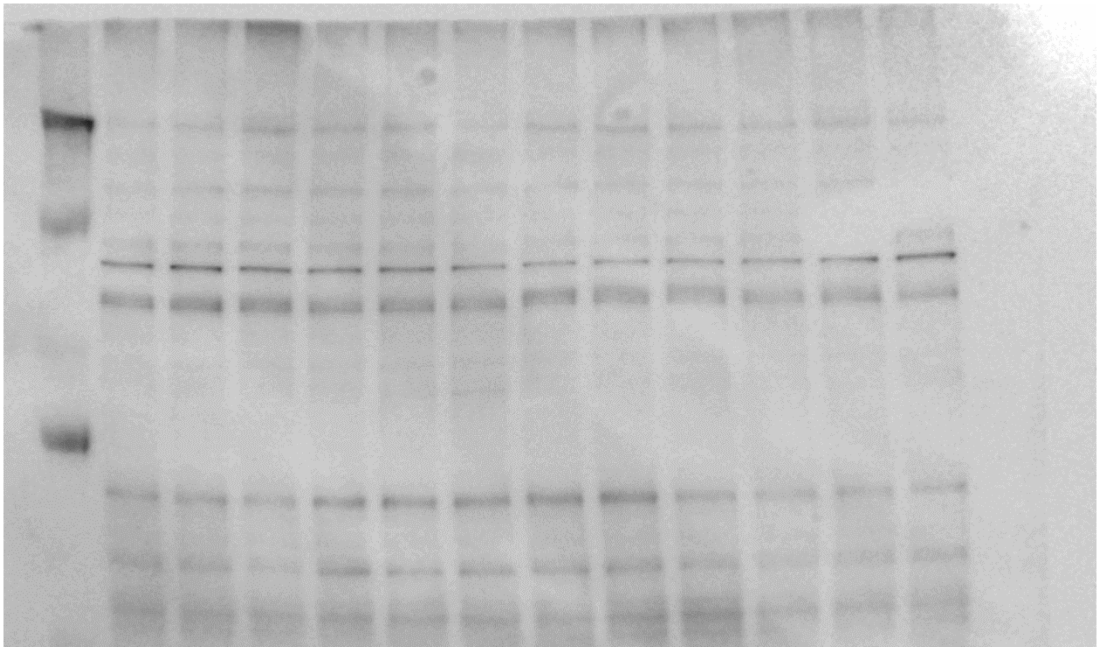

Figure 5A.

IB: Vinculin

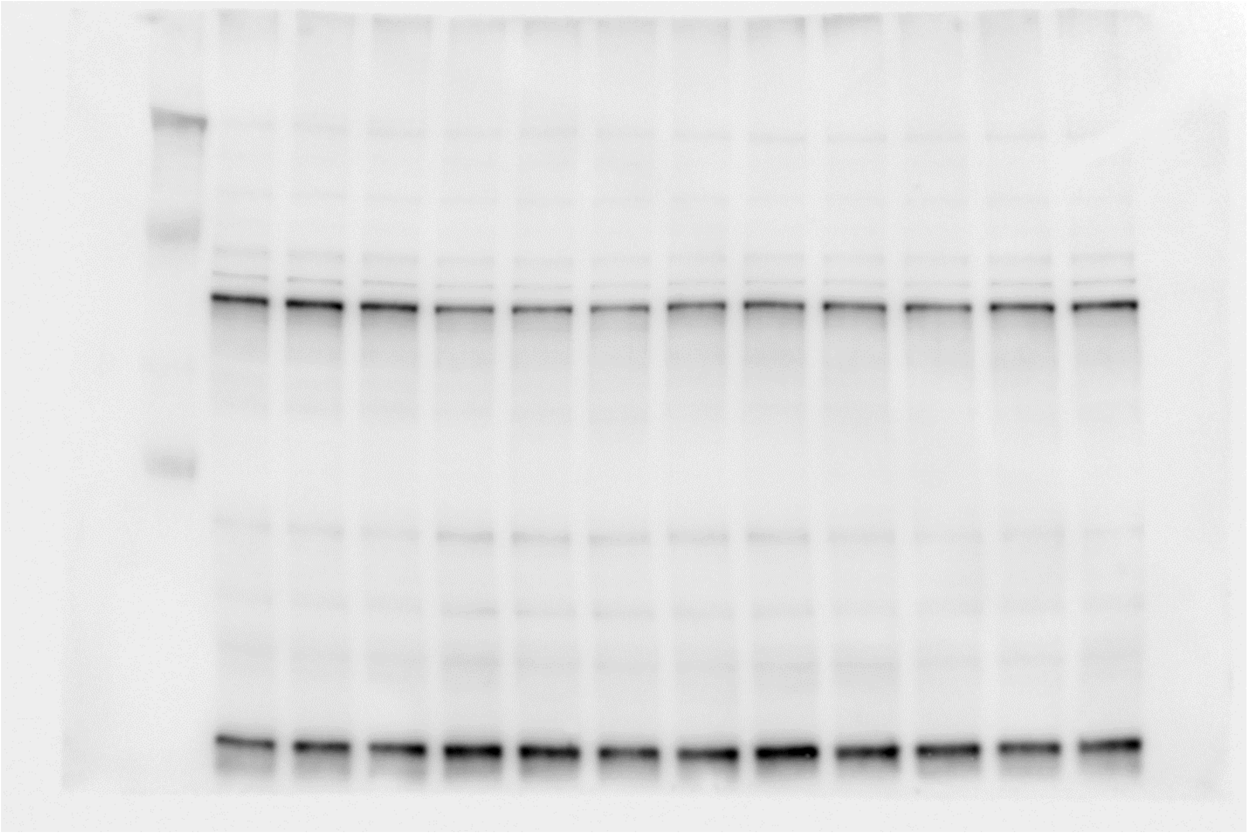

Figure 5C

IB: pMek

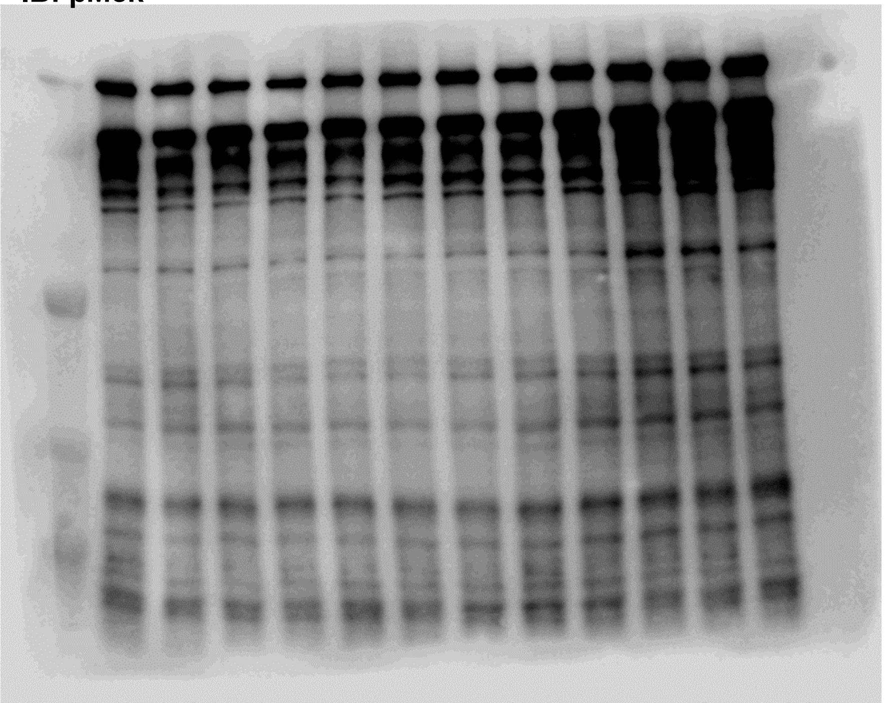

IB: Mek

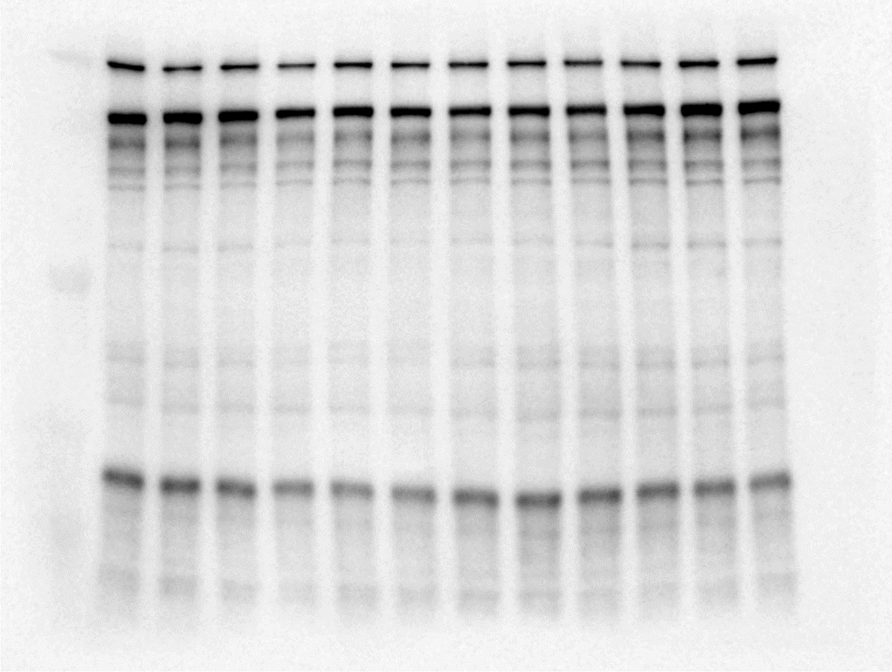

Figure 5C

IB: p-p44/42

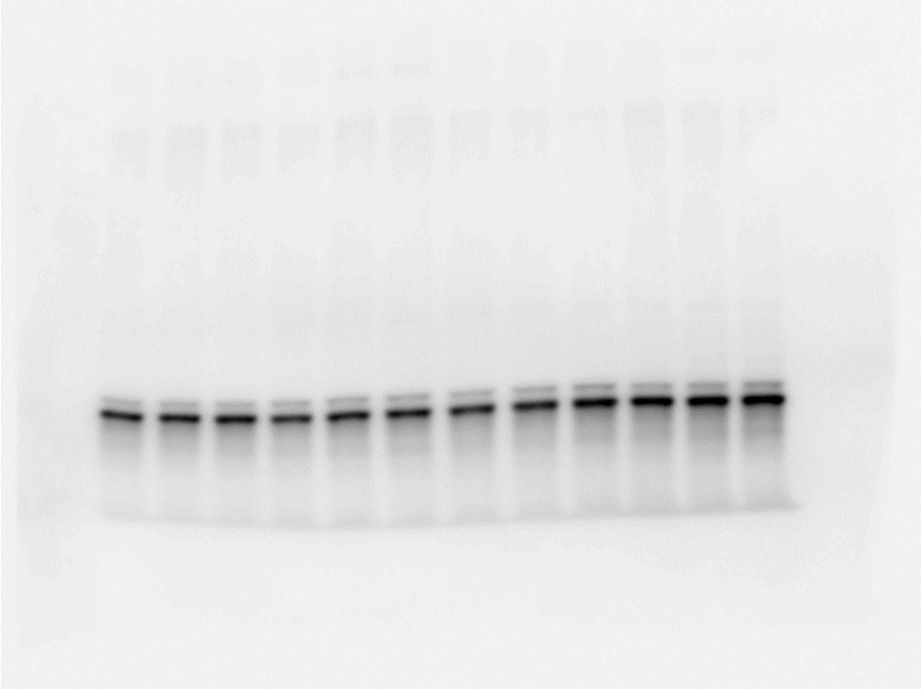

IB: p44/42

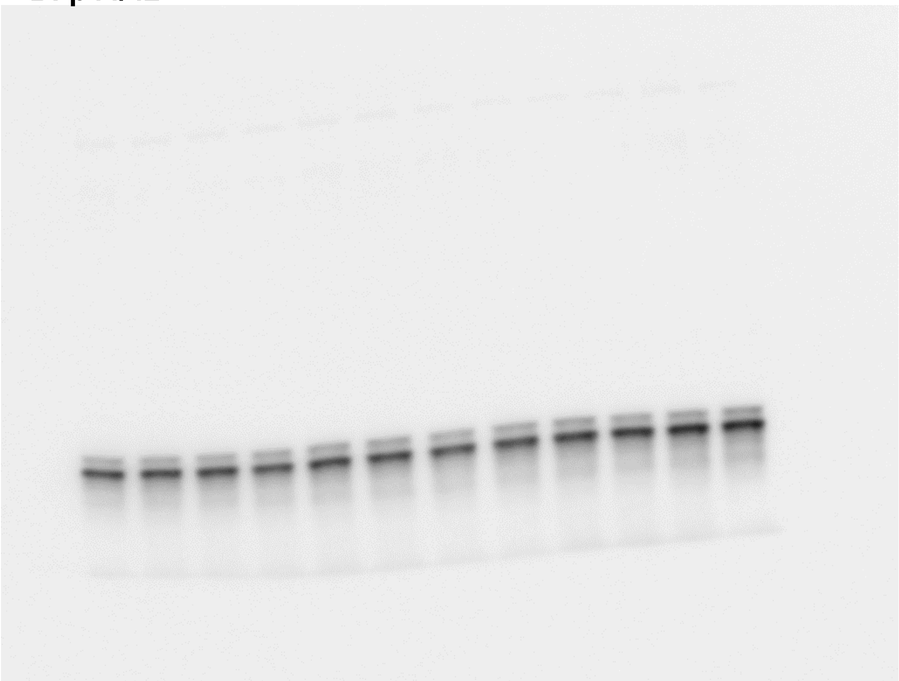

Figure 5C

IB: Vinculin

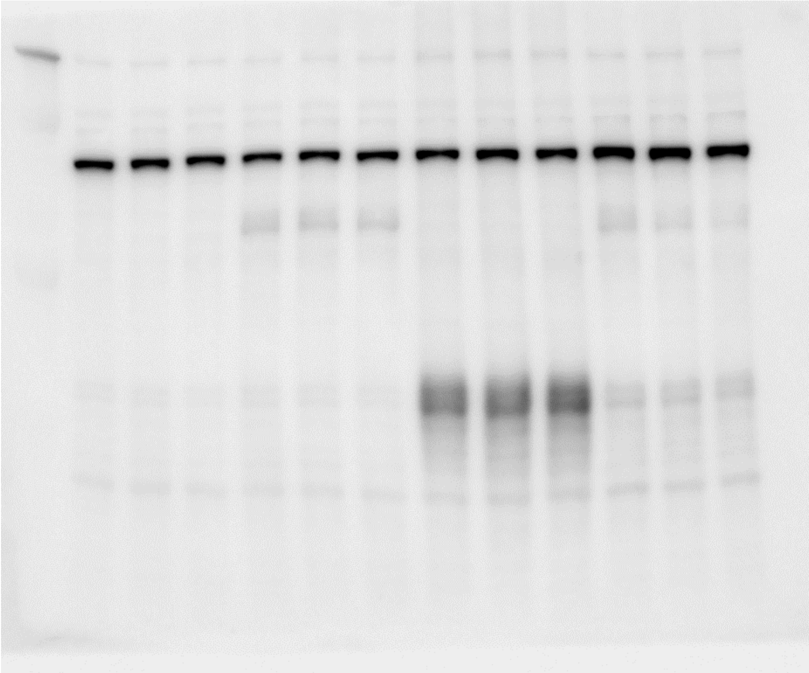

Figure 6A

IB: KRAS IB: Vinculin IB: hPRLr-ECD

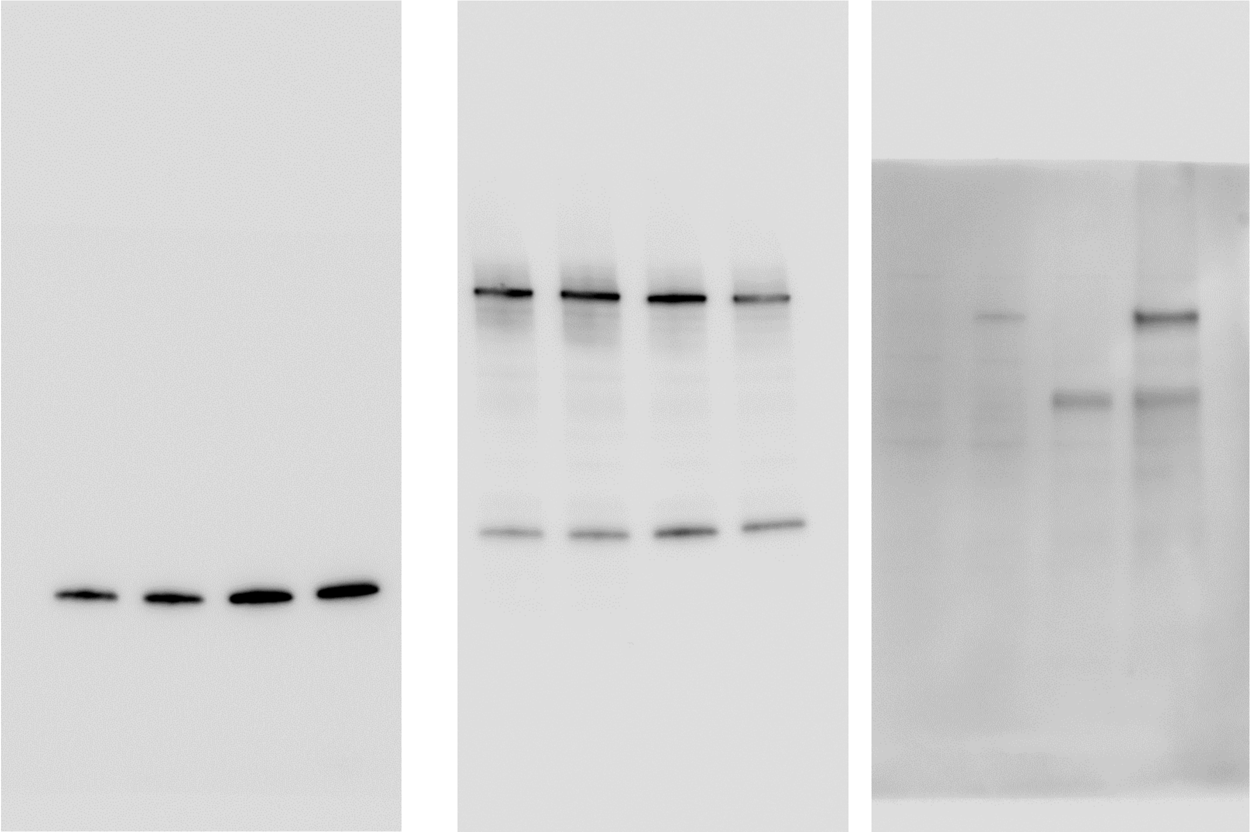

Supplementary Figure 3A

IB: hPRLr-ECD

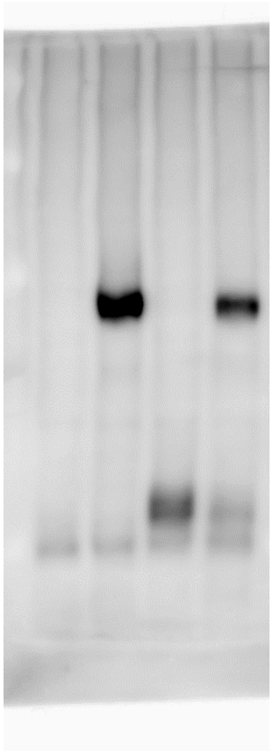

IB: Vinculin

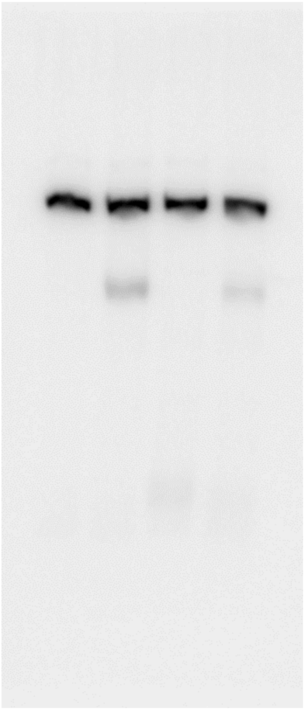

Supplementary Figure 4A

IB: Vinculin

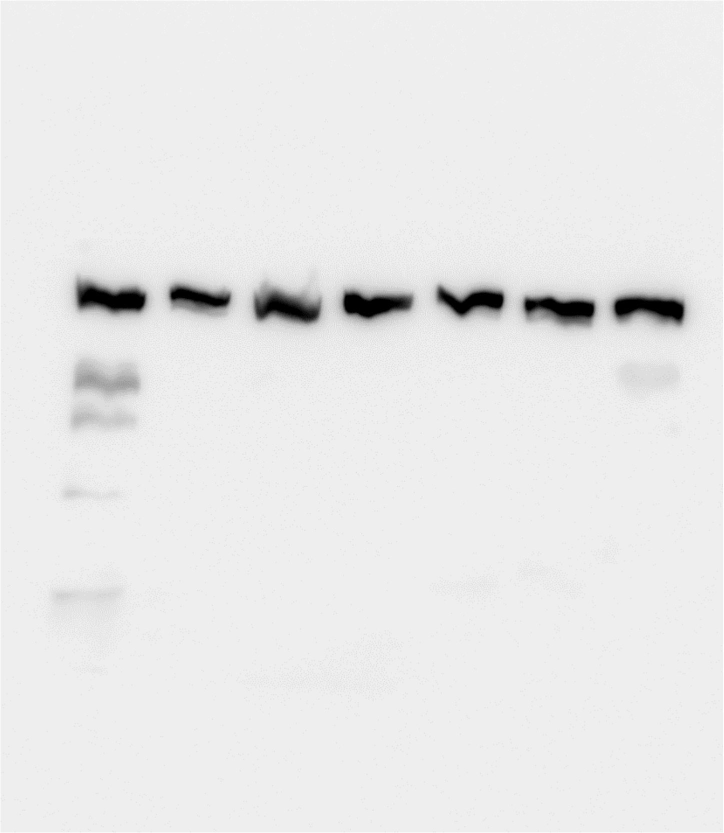

IB: hPRLr-ECD

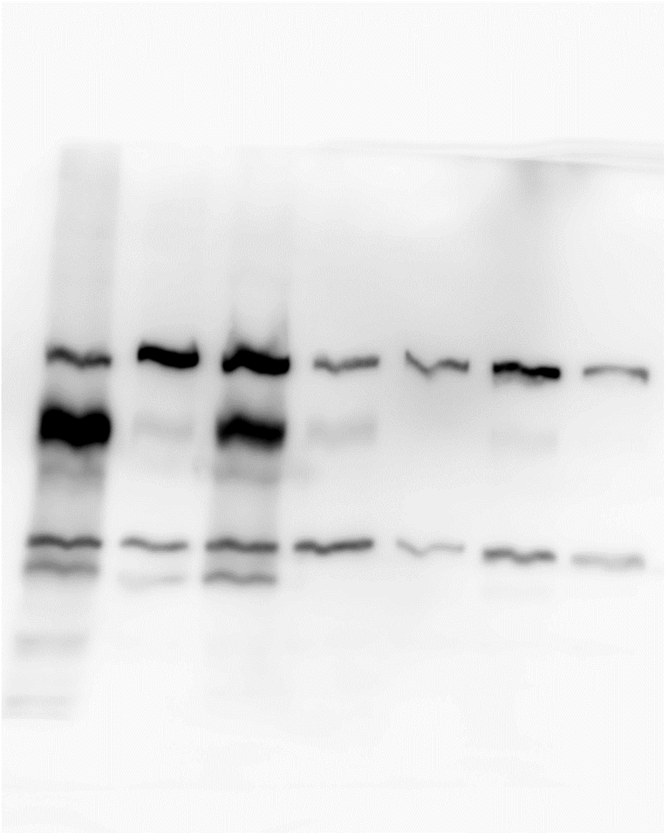

Supplementary Figure 4B

IB: hPRLrI

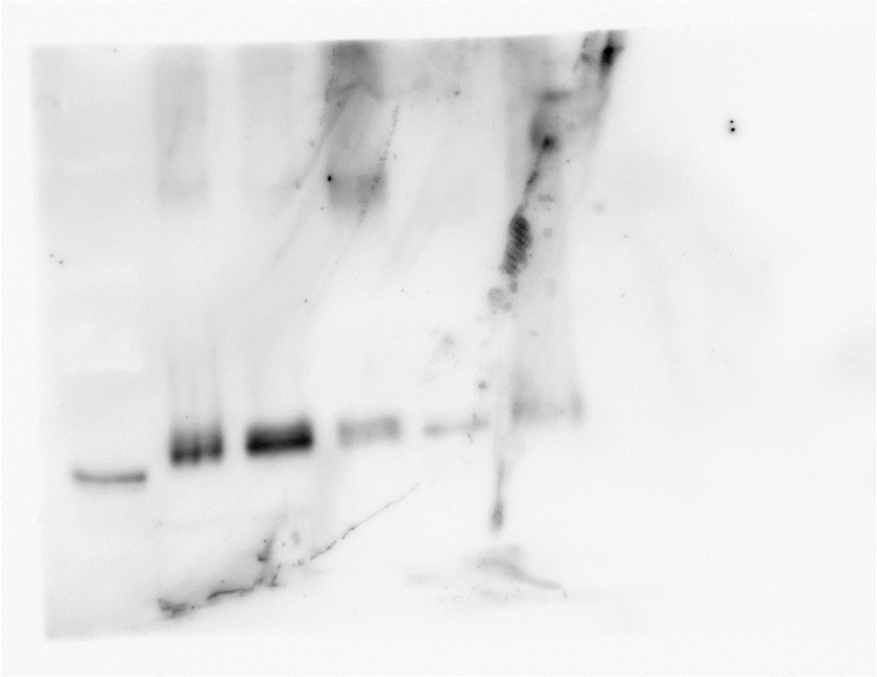

IB: hPRLr-ECD

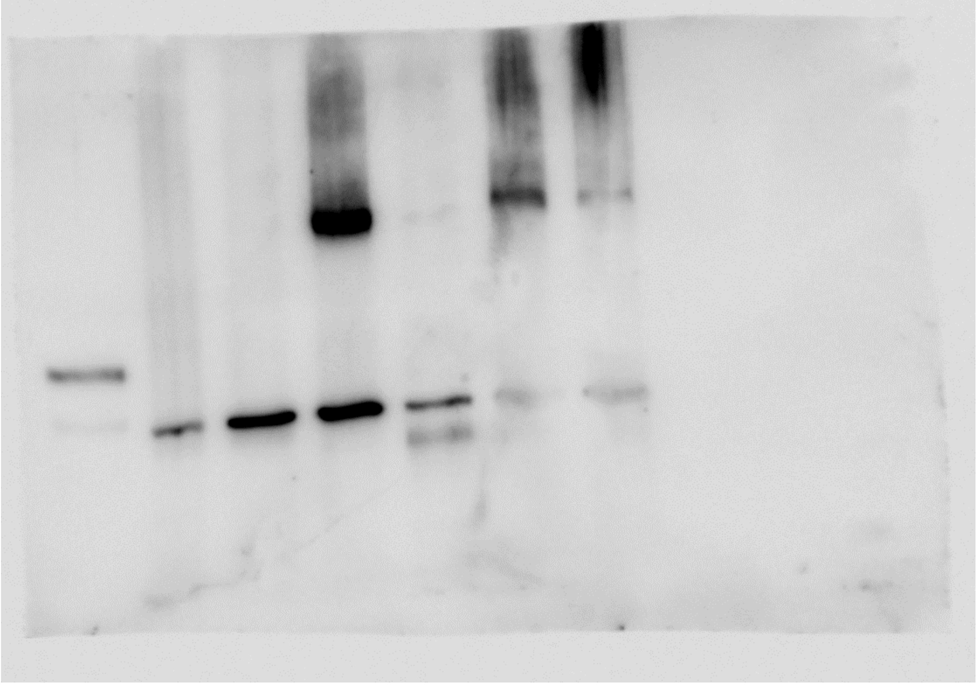

Supplementary Figure 4B

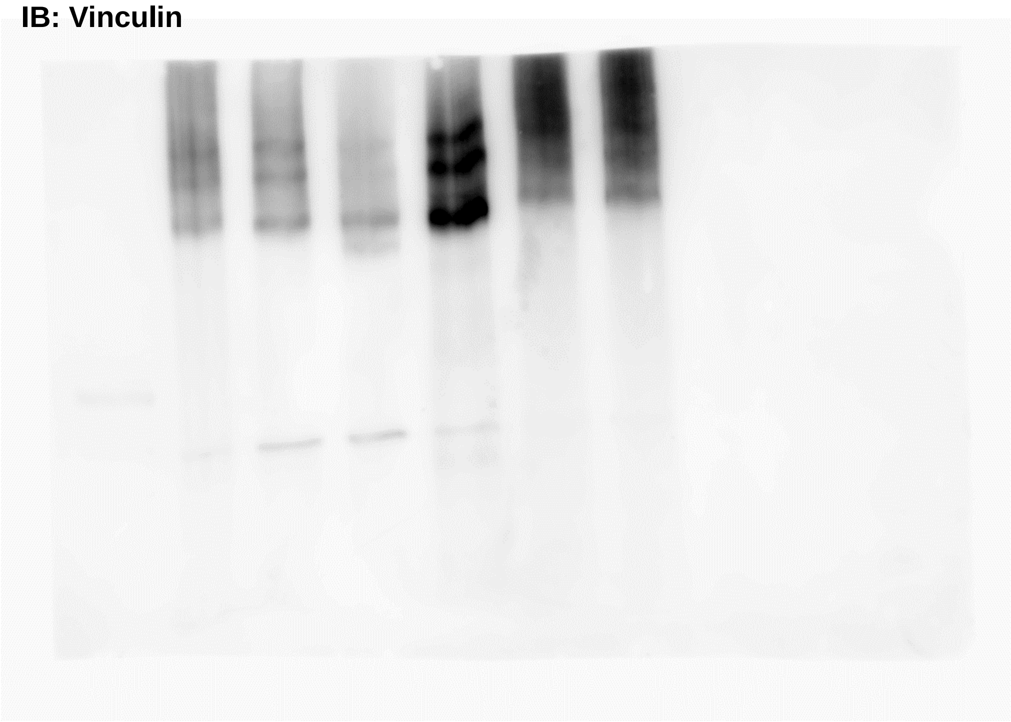

Supplementary Figure 4C

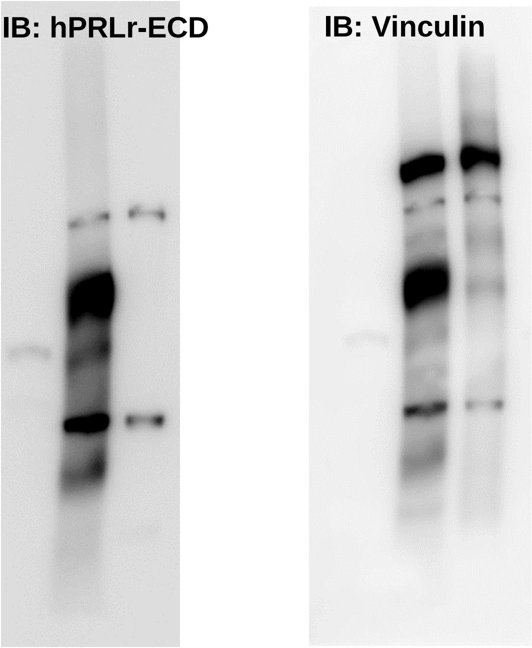

Supplementary Figure 5A

IB: hPRLr-ECD

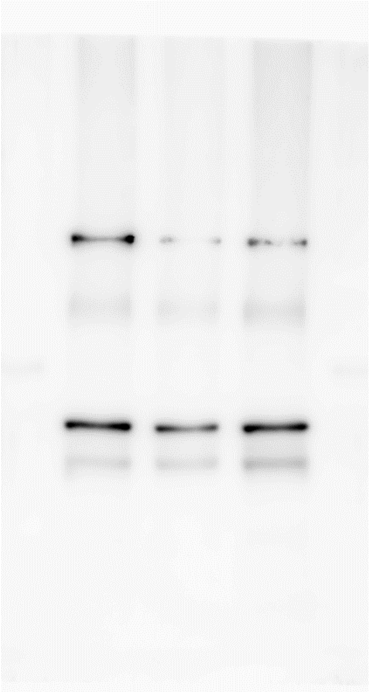

IB: Vinculin

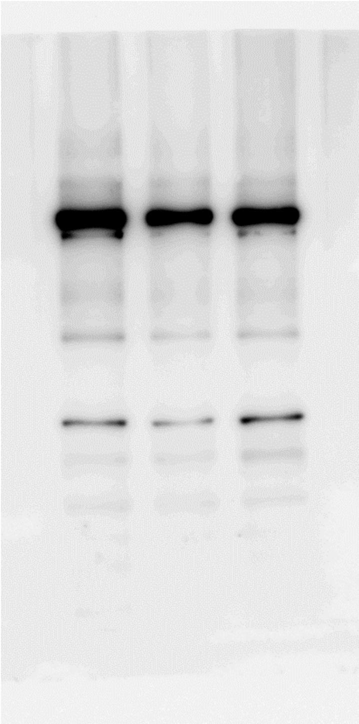

Supplementary Figure 6A

IB: hPRLr-ECD

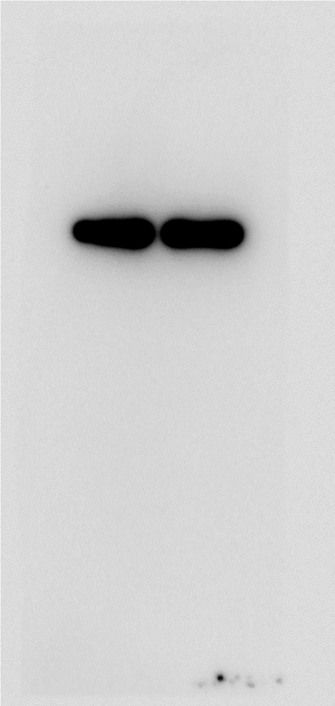

IB: hPRLrI

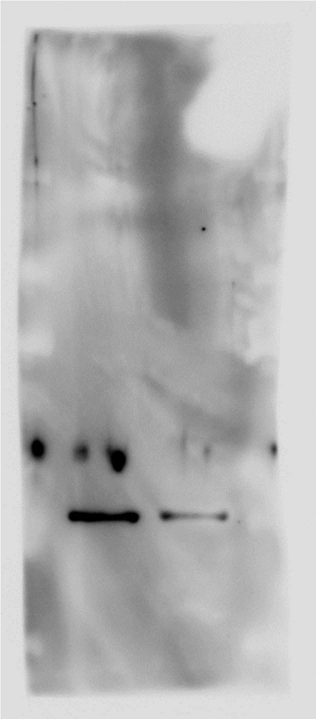

IB: Vinculin

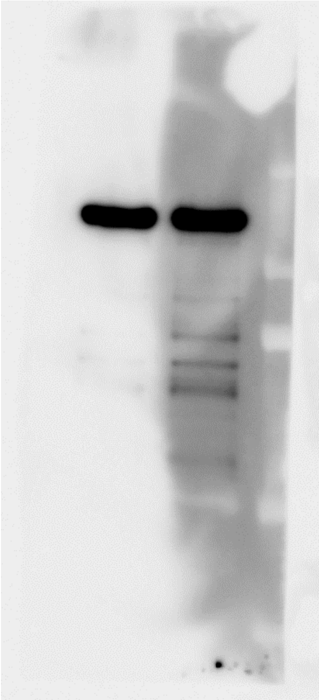

Supplementary Figure 6A

IB: hPRLr-ECD

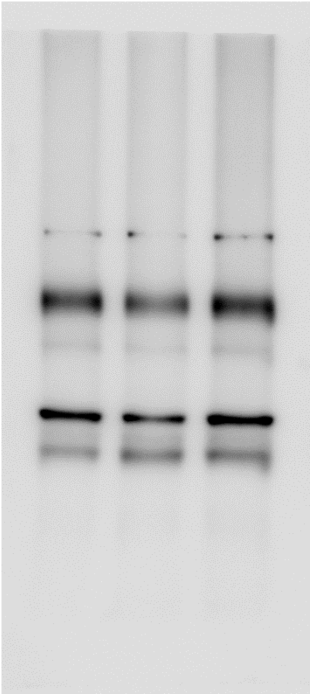

IB: Vinculin

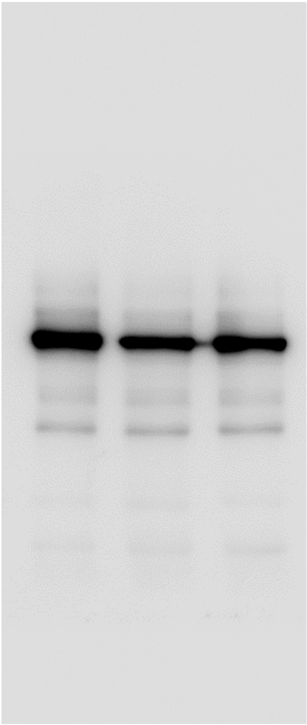

Supplementary Figure 7A

IB: hPRLr-ECD

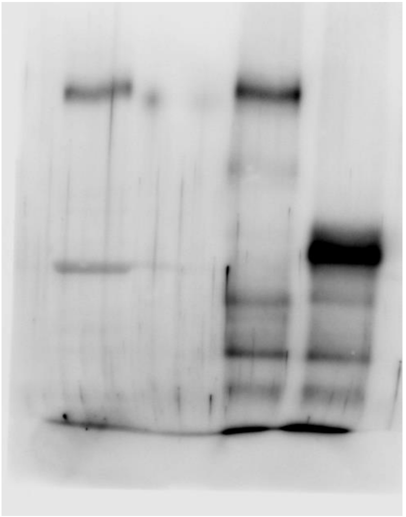

IB: hPRLrI

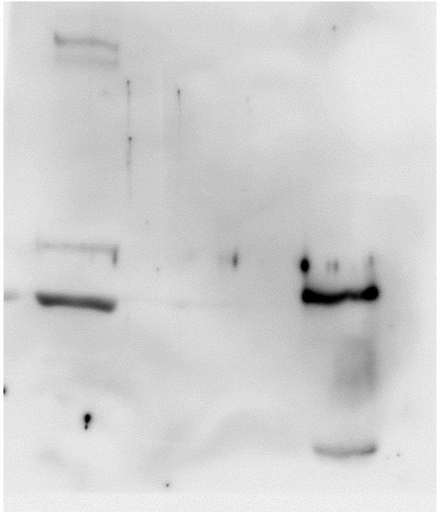

Supplementary Figure 7A

IB: Vinculin

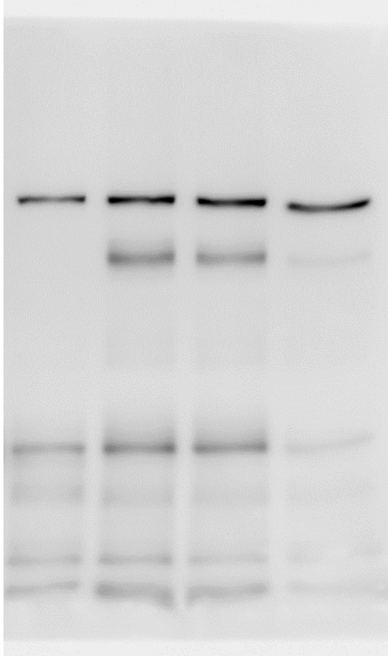

IB: Vinculin

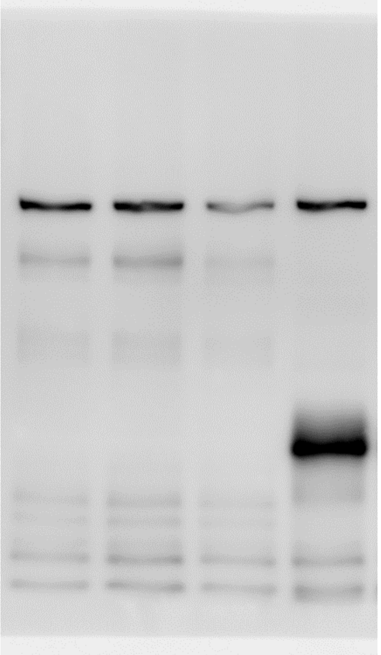

Supplementary Figure 7B

IB: hPRLrI -/+ blocking peptide

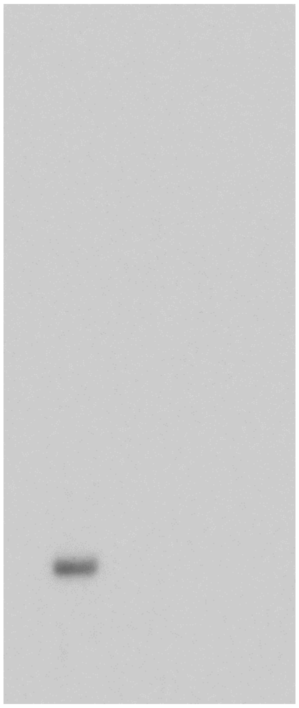

IB: Vinculin

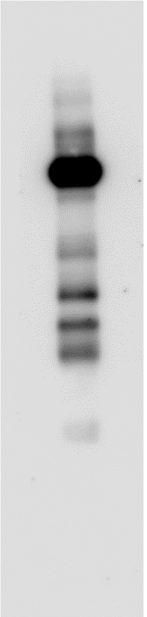

IB: Vinculin

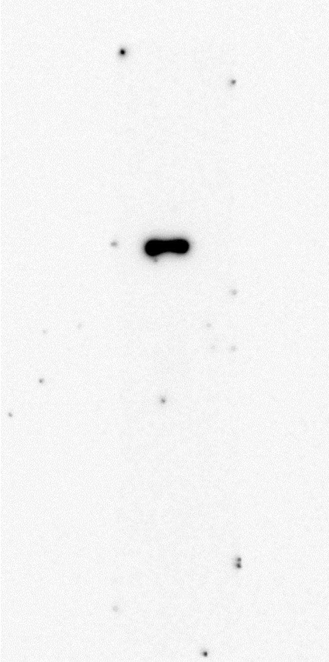

Supplement: Supplementary file 1 — Supplementary Information [file 41523_2021_243_MOESM1_ESM.pdf]
