# Supplementary material for: The human intermediate prolactin receptor is a mammary proto-oncogene
Source: NPJ Breast Cancer. 2021 Mar 26;7:37. doi: 10.1038/s41523-021-00243-7 (PMC7997966; doi:10.1038/s41523-021-00243-7)
Supplement: Supplementary file 2 — Reporting Summary Checklist [file 41523_2021_243_MOESM2_ESM.pdf]

nature research

Corresponding author(s): Charles V. Clevenger

Last updated by author(s): Mar 3, 2021

Reporting Summary

Nature Research wishes to improve the reproducibility of the work that we publish. This form provides structure for consistency and transparency in reporting. For further information on Nature Research policies, see our Editorial Policies and the Editorial Policy Checklist.

Statistics

For all statistical analyses, confirm that the following items are present in the figure legend, table legend, main text, or Methods section.

n/a	Confirmed	
☐	☒	The exact sample size (*n*) for each experimental group/condition, given as a discrete number and unit of measurement
☐	☒	A statement on whether measurements were taken from distinct samples or whether the same sample was measured repeatedly
☐	☒	The statistical test(s) used AND whether they are one- or two-sided<br>*Only common tests should be described solely by name; describe more complex techniques in the Methods section.*
☐	☒	A description of all covariates tested
☐	☒	A description of any assumptions or corrections, such as tests of normality and adjustment for multiple comparisons
☐	☒	A full description of the statistical parameters including central tendency (e.g. means) or other basic estimates (e.g. regression coefficient) AND variation (e.g. standard deviation) or associated estimates of uncertainty (e.g. confidence intervals)
☒	☐	For null hypothesis testing, the test statistic (e.g. *F*, *t*, *r*) with confidence intervals, effect sizes, degrees of freedom and *P* value noted<br>*Give P values as exact values whenever suitable.*
☒	☐	For Bayesian analysis, information on the choice of priors and Markov chain Monte Carlo settings
☒	☐	For hierarchical and complex designs, identification of the appropriate level for tests and full reporting of outcomes
☒	☐	Estimates of effect sizes (e.g. Cohen's *d*, Pearson's *r*), indicating how they were calculated

Our web collection on statistics for biologists contains articles on many of the points above.

Software and code

Policy information about availability of computer code

Data collection	The bioinformatics tools used in this study are: SamTools, STAR, Integrated Genome Browser, R (DESeq2), GSEA (Broad)
Data analysis	All statistical analyses were completed using PRISM or DESeq2

For manuscripts utilizing custom algorithms or software that are central to the research but not yet described in published literature, software must be made available to editors and reviewers. We strongly encourage code deposition in a community repository (e.g. GitHub). See the Nature Research guidelines for submitting code & software for further information.

Data

Policy information about availability of data

All manuscripts must include a data availability statement. This statement should provide the following information, where applicable:

- Accession codes, unique identifiers, or web links for publicly available datasets
- A list of figures that have associated raw data
- A description of any restrictions on data availability

All relevant scripts and data can be found at: https://github.com/AmyOlex/hPRLrI_TCGA

Field-specific reporting

Please select the one below that is the best fit for your research. If you are not sure, read the appropriate sections before making your selection.

☒ Life sciences ☐ Behavioural & social sciences ☐ Ecological, evolutionary & environmental sciences

For a reference copy of the document with all sections, see nature.com/documents/nr-reporting-summary-flat.pdf

Life sciences study design

All studies must disclose on these points even when the disclosure is negative.

Sample size	n=3 for all experiments, as we reasoned this would be sufficient to assess variation in our experimental approach(es) and determine statistical significance
Data exclusions	NA
Replication	In vitro studies were replicated with two different cell lines (MCF10A vs MCF10AT, T47D vs MCF7)
Randomization	NA
Blinding	NA

Reporting for specific materials, systems and methods

We require information from authors about some types of materials, experimental systems and methods used in many studies. Here, indicate whether each material, system or method listed is relevant to your study. If you are not sure if a list item applies to your research, read the appropriate section before selecting a response.

Materials & experimental systems

n/a	Involved in the study	
☐	☒	Antibodies
☐	☒	Eukaryotic cell lines
☒	☐	Palaeontology and archaeology
☐	☒	Animals and other organisms
☒	☐	Human research participants
☒	☐	Clinical data
☒	☐	Dual use research of concern

Methods

n/a	Involved in the study	
☒	☐	ChIP-seq
☒	☐	Flow cytometry
☒	☐	MRI-based neuroimaging

Antibodies

Antibodies used	hPRLr ECD (35-9200, Invitrogen, 1:1,000), pY-Stat5a (9359S, Cell Signaling, 1:1,000), Stat5a (sc-1081, Santa Cruz Biotechnology, 1:1,000), pY-Jak2 (3776S, Cell Signaling, 1:500), Jak2 (3230S, Cell Signaling, 1:500), p-p44/42 (9101S, Cell Signaling, 1:1,000), p44/42 (9102S, Cell Signaling, 1:1,000), pS-Mek (9121S, Cell Signaling, 1:1,000), Mek (9122S, Cell Signaling, 1:1,000), KRAS (14412S, Cell Signaling, 1:1,000), hPRLrI (New England Peptide, 1:5,000), pS349-hPRLr (Serge Y. Fuchs, M.D., Ph.D., University of Pennsylvania, 1:100), Vinculin (MCA465GA, Bio-Rad, 1:1,000)
Validation	Validation for all commercially-purchased antibodies was performed by the manufacturer. Validation for the custom-generated hPRLrI antibody can be found in Supplementary Figure 7. Validation for the hPRLr-pS349 antibody can be found in the following publication: Li, Y., Clevenger, C., Minkovsky, N. et al. Stabilization of prolactin receptor in breast cancer cells. Oncogene 25, 1896–1902 (2006).

Eukaryotic cell lines

Policy information about cell lines

Cell line source(s)	ATCC
Authentication	ATCC
Mycoplasma contamination	All cell lines used were routinely checked for mycoplasma contamination.
Commonly misidentified lines (See ICLAC register)	*Name any commonly misidentified cell lines used in the study and provide a rationale for their use.*

Animals and other organisms

Policy information about studies involving animals; ARRIVE guidelines recommended for reporting animal research

Laboratory animals	Mice, NSG, female
Wild animals	NA
Field-collected samples	NA
Ethics oversight	Virginia Commonwealth University Institutional Animal Care and Use Committee (IACUC) together with the Division of Animal Resources (DAR)

Note that full information on the approval of the study protocol must also be provided in the manuscript.
